# SOX9 in organogenesis: shared and unique transcriptional functions

**DOI:** 10.1007/s00018-022-04543-4

**Published:** 2022-09-17

**Authors:** Zhenhua Ming, Brittany Vining, Stefan Bagheri-Fam, Vincent Harley

**Affiliations:** 1 Sex Development Laboratory, Hudson Institute of Medical Research, PO Box 5152, Melbourne, VIC 3168 Australia; 2 Department of Molecular and Translational Science, Monash University, Melbourne, VIC 3800 Australia

**Keywords:** SOX, Transcription factor, Organ development, Gene regulation, Cell specification/differentiation, Campomelic dysplasia

## Abstract

The transcription factor SOX9 is essential for the development of multiple organs including bone, testis, heart, lung, pancreas, intestine and nervous system. Mutations in the human *SOX9* gene led to campomelic dysplasia, a haploinsufficiency disorder with several skeletal malformations frequently accompanied by 46, XY sex reversal. The mechanisms underlying the diverse SOX9 functions during organ development including its post-translational modifications, the availability of binding partners, and tissue-specific accessibility to target gene chromatin. Here we summarize the expression, activities, and downstream target genes of SOX9 in molecular genetic pathways essential for organ development, maintenance, and function. We also provide an insight into understanding the mechanisms that regulate the versatile roles of SOX9 in different organs.

**Supplementary Information:**

The online version contains supplementary material available at 10.1007/s00018-022-04543-4.

## Introduction

Mammalian embryogenesis is a complicated process; a cross-talking network of instructions for specialized developmental processes. Many transcription factors are essential for multiple developmental pathways, or to direct a large variety of processes. One such transcription factor is sex-determining region Y (SRY)-box 9 (SOX9)—a member of the SOX (SRY-type HMG box) family of transcription factors. These all harbor a high mobility group (HMG) box DNA-binding domain, first identified in SRY. Twenty SOX proteins have since been identified in mice and humans, and are grouped into nine subfamilies (A, B1, B2, C–H) depending on the structural homology outside of the HMG box [1]. Heterozygous mutations in *SOX9* lead to the human disorder campomelic dysplasia (CMPD, OMIM# 114, 290) characterized by skeletal dysplasia and variable 46, XY sex reversal [2, 3]. Studies of *Sox9*-deficient mice, and SOX9 function in other species, have demonstrated its diverse roles during the development in multiple tissues and organs. These include cartilage [4], testis [5], nervous system [6], retina [7], lung [8], heart valve [9], pancreas [10], bile duct [11], intestine [12], prostate [13], and hair follicle [14]. In this review, we describe common and unique functions of SOX9 among tissues and organs, and the varied mechanisms through which SOX9 acts. Overall, this review comprehensively highlights the essential role that SOX9 plays in mammalian embryogenesis and organogenesis, thereby providing insights into the role this transcription factor plays to both initiate and maintain regulatory processes.

## SOXE, SOX9, and their functional domains

**Fig. 1:**
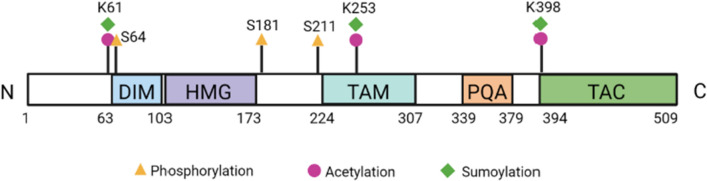
Schematic structure of the human SOX9 protein. The dimerization domain (DIM) precedes the HMG box. Two transactivation domains are located in the middle (TAM) and at the C-terminus (TAC). The proline, glutamine and alanine (PQA)-rich domain is required for transactivation. Phosphorylation of serine (S) residues, acetylation and sumoylation of lysine (K) residues are highlighted

The *SOX9/Sox9* gene lies on human chromosome 17q and mouse chromosome 11q. *SOX9* is located in a gene desert containing long-range spatiotemporal specific enhancers. The human SOX9 protein comprises 509 amino acids and consists of a HMG box, a dimerization domain (DIM), two transactivation domains located in the middle (TAM) and the C-terminus (TAC) of the protein, and a proline/glutamine/alanine (PQA)-rich domain (Fig. 1).

SOX9, SOX8 and SOX10 form the SOXE subgroup, sharing homology in the HMG, DIM, and TAM and TAC domains, with two nuclear localization signals (NLS) and one nuclear export signal (NES). The HMG domain facilitates sequence-specific DNA binding and binds to the minor groove of DNA, inducing DNA bending by forming an L-shaped complex. The consensus DNA-binding motif of SOX9 is AGAACAATGG, with AACAAT being the core-binding element, and flanking 5′ AG and 3′ GG nucleotides specific to SOX9 [15].

SOX9 is capable of homodimerization through the DIM domain; this is required for DNA binding and transactivation of cartilage-specific genes [16, 17]. SOXE proteins heterodimerize via the DIM domain of one protein and the HMG box of the other protein [18]. In contrast, SOX9 functions as a monomer in testicular Sertoli cells [16]. Active SOX9-binding dimer motifs in regulatory regions of target genes are cell-type specific: SOX9 dimerizes on palindromic composite DNA motifs separated by 3–5 nucleotides in melanoma cells [18] and chondrocytes [19], whereas no enrichment of palindromic sequences is observed in hair follicle stem cells [20].

SOX9 transactivation domains interact with transcriptional co-activators or basal transcriptional machinery components. The transactivation domain at the C-terminus (TAC) physically interacts with MED12 (mediator complex subunit 12), CBP/p300 (CREB binding protein/E1A binding protein p300), TIP60 (Tat interactive protein-60), and WWP2 (WW domain containing E3 ubiquitin protein ligase 2), enhancing transcriptional activity of SOX9 [21–24]. The TAC domain is required for inhibition of β-catenin during chondrocyte differentiation [25]. The transactivation domain in the middle (TAM) synergizes with the TAC domain to activate cartilage-specific genes in vitro [26].

SOX9 also contains a unique PQA-rich domain that enhances transactivation in vitro [27] but lacks autonomous transactivation capability [26]. Deletion of the PQA-rich domain reduced SOX9’s capacity to transactivate a reporter plasmid with tandemly repeated SOX9 binding sites [27]. These domains thus allow SOX9 to enact different roles within the complex molecular pathways in which it plays such a crucial part.

## SOX9 in organ and tissue development

SOX9 plays an important role in organogenesis during mammalian embryo development. The expression profile, function, and target genes that SOX9 directly binds to and activates during organ development will be discussed. A diagrammatic summary of cell-type specific expression of SOX9 during the development of cartilage, testis, nervous system, retina, lung, heart valve, pancreas, bile duct, intestine, prostate, and hair follicle is shown in Supplementary Fig. 1. The functions and target genes activated by SOX9 are summarized in Table 1.

**Table 1:** SOX9 roles and targets in organs and tissues

Organs/tissues	SOX9 roles	SOX9 targets
Cartilage	Chondrogenic mesenchymal condensation [34]	*Fam101a* [35], *Myh14* [35], *Sema3c* [35], *Sema3d* [35]
	Chondrocyte survival, differentiation, proliferation [33]	*Sox5* [33, 44], *Sox6* [33, 44], *COL2A1* [37]/*Col2a1* [33, 44], *Acan* [33, 44], *Comp* [33, 176]
	Chondrocyte hypertrophy [40, 42]	*Col10a1* [40]
	ECM components regulation [33, 44]	all major cartilage ECM genes [33, 44]
	Cartilage TFs regulation [33, 44]	*Sox5* [33, 44], *Sox6* [33, 44], *Sox9* [33, 44]
Testis	Sertoli cell differentiation [5, 52]	*SOX9* [122]/*Sox9* [5, 52]
	Repression of the ovarian pathway [54, 56]	*Foxl2* [56]
	Testis TFs regulation [52, 55, 56]	*Sox8* [52, 55, 56], *Sox10* [52, 55, 56]
	*Sox9* expression maintenance [54, 59, 60, 120]	*Ptgds* [59, 60], *Fgf9* [54], *Sox9* [120]
	FLC and PMC differentiation, fate of germ cell [5, 52, 56, 61]	*Cyp26b1* [56, 61], *Dhh* [56]
	Müllerian ducts degeneration [5]	*AMH* [169]/*Amh* [55, 65]
	Adult fertility maintenance [57]	*ETV5* [67]/*Etv5* [67], *Gdnf* [55–57]
Nervous system	Induction and maintenance of neural stem cells [6]	–
	Glial specification [6, 69]	–
	Astrocyte differentiation [69]	*Nfia* [70], *Apcdd1* [70], *Mmd2* [70], *Zcchc24* [70], *Nfe2l1* [71]
	Oligodendrocyte differentiation [70]	*Pdgfra* [73]
Retina	Müller glial cell specification [75]	–
	RPE differentiation and maturation [76, 77]	*RPE65* [77]/*Rpe65* [77], *RLBP1* [77], *RGR* [77]/*Rgr* [77]
	Choroid development [76]	*ANGPTL4* [76]/*Angptl4* [76]
Lung	Branching morphogenesis [8, 80]	–
	Distal lung progenitor maintenance [80]	–
	Alveolar epithelium migration, polarity [8, 80]	–
	Alveolar ECM production [8]	*Col2a1* [8], *Laminin* [8]
Heart	Progenitor cell proliferation [82]	*Cops5* [82], *Junb* [82], *Fosl1* [82], *Fosl2* [82], *Fos* [82], *Srpk2* [82], *Akt2* [82], *Eed* [82], *Hdac1* [82], *Hdac2* [82]
	ECM components regulation [82]	*Hapln1* [82], *Acan* [82], *Col2a1* [82], *Eln* [82], *Postn* [82]
	Heart valve TFs regulation [82]	*Mecom* [82], *Lef1* [82], *Pitx2* [82], *Hand2* [82], *Twist1* [82], *Sox4* [82]
Pancreas	Pancreatic progenitor maintenance [10, 95]	*Fgfr2b* [95]
	Endocrine differentiation [92]	*Neurog3* [92, 98]
	Repression of hepatic and intestinal genes [95, 96, 100]	*CDX2* [100]/*Cdx2* [100], *ONECUT-2* [100]/*Onecut-2* [100], *NKX6-3* [100]/*Nkx6.3* [100]
	Primary cilia formation [98]	–
	Pancreas TFs regulation [100]	*PTF1A* [100], *PAX6* [100], *NEUROG3* [100]
Bile duct	Bile duct maturation [11, 102]	–
	Cholangiocyte polarity [102]	*Spp1* [102]
	Primary cilia formation [102]	–
Intestine	Progenitor maintenance [107]	*CDX2* [107], *MUC2* [107]
	Differentiation of Paneth cells and goblet cells [12, 106]	
Prostate	Ventral prostate proliferation [13]	*Shh* [13], *Fgfr2* [13], *Spry2* [13]
	Anterior prostate differentiation [13]	–
	Prostatic bud elongation [109]	*Cdc42* [109], *Barx2* [109], *Cxcr4* [109], *Col14a1* [109]
Hair follicle	Stem cell specification [114]	–
	Matrix cell proliferation [14]	–
	Adult HF-SCs maintenance [14, 20]	–
	Adult HF TFs regulation [20]	*Lhx2* [20], *Nfatc1*[20], *Tcf7l2* [20]
	Adult HF ECM components regulation [20]	*Timp2* [20], *Wfdc3* [20], *Col8a2* [20]

Genes in upper case refer to human in vitro studies, while genes in lower case refer to in vivo rodent studies. Dash line symbol (–) denotes currently unidentified SOX9 targets

### Cartilage

#### Chondrogenesis and SOX9 expression

Endochondral ossification creates fetal bone tissue from a cartilage template and later forms bones of the axial and appendicular skeleton (Fig. S1A). At the beginning, mesenchymal cells condense and migrate toward the sites of future bone. Bipotential osteochondroprogenitors commit to the chondrocytic fate, differentiate into prechondrocytes and deposit cartilage matrix. The early chondrocytes in the cartilage anlagen undergo proliferation, maturation, cell cycle exit, and hypertrophy. Between the epiphyses and bone shaft, the cartilage growth plate is composed of four zones of activity, allowing longitudinal bone growth.

The resting or reserve zone contains small round chondrocytes, serving as progenitor-like cells for subsequent proliferation and differentiation. The proliferating zone contains proliferating chondrocytes organized into longitudinal columns. The prehypertrophic zone comprises maturing chondrocytes which undergo cell cycle arrest and initiate expression of the hypertrophic markers Indian hedgehog (*Ihh*) [28] and collagen type 10 gene (*Col10a1*) [29]. The hypertrophic zone is composed of massively expanded chondrocytes [30]. These hypertrophic chondrocytes undergo terminal differentiation and remodeling, mineralize the cartilage matrix, and may undergo apoptosis or transdifferentiate into osteoblasts [31, 32].

During chondrogenesis, SOX9 is expressed in condensed mesenchymal cells and differentiated chondrocytes of the cartilage growth plate, except in hypertrophic chondrocytes in which its expression is shut off [33]. In the developing limbs, SOX9 is abundant in mesenchymal condensation at embryonic day E11.5–E12.5 [4]. Most cartilage is well formed by E15.5, and SOX9 is expressed in the resting, proliferating and prehypertrophic chondrocytes within the growth plates of the long bones. SOX9 is a master regulator in chondrogenesis and is required for successive steps of chondrocyte differentiation which are described in detail below.

#### Chondrogenic mesenchymal condensation and survival of mesenchymal cells

During chondrogenesis, SOX9 is required for chondrogenic mesenchymal condensations. In mouse embryo chimeras*, Sox9*^−/−^ cells are excluded from mesenchymal condensations and fail to express chondrocyte-specific markers *Col2a1*, *Col9a2*, *Col11a2*, and *Acan* (aggrecan) [34]. Accordingly, inactivation of *Sox9* in all mesenchymal cells of the limb buds results in complete loss of cartilage and bone in limbs [33, 34]. At E13.5, the mesenchymal cells in *Sox9*^−/−^ limb buds display extensive apoptosis, suggesting that SOX9 is necessary for their survival [33]. SOX9 likely controls prechondrocyte condensation by upregulating several genes that are crucial for actin cytoskeleton assembly, homotypic cell–cell adhesion and heterotypic cell–cell repulsion [35]. These genes include *Fam101a* (filamin-interacting protein), *Myh14* (myosin heavy chain 14), *Sema3c* (semaphorin 3c) and *Sema3d*, which are directly transactivated by SOX9 via binding to their enhancers.

#### Chondrocyte differentiation and proliferation

Condensed prechondrocytes differentiate into early-stage chondrocytes, which start to produce cartilage extra-cellular matrix (ECM). If *Sox9* is inactivated in mice from E13.5, after mesenchymal condensations, severe chondrodysplasia is observed as most condensed mesenchymal cells fail to differentiate into chondrocytes [33]. The expression of *Sox5*, *Sox6* and extracellular matrix genes *Col2a1*, *Acan*, and *Comp* (cartilage oligomeric matrix protein) are markedly down-regulated in metacarpals of these mice at E15.5. Moreover, SOX9 is necessary for chondrocyte proliferation as the size of proliferating zones in these mice is dramatically reduced in the absence of *Sox9* [33].

During early chondrogenesis, SOX9 cooperates with SOX5/SOX6 to activate expression of target genes, such as *Col2a1* and *Acan* by binding to their enhancers [36]. The three SOX proteins associate with insulin-like growth factor-I (IGF-I), transcription factors Sp1/Sp3, and euchromatin-associated factors CBP/p300 and TIP60 to increase *COL2A1* transcription by interacting with its enhancer region [37]. In addition, the SOX trio physically interacts with the Runt domain transcription factor (RUNX1) to enhance activation of chondrocyte-specific genes thereby increasing cartilage matrix production [38]. Moreover, in non-hypertrophic chondrocytes, GLI2 and GLI3 functionally interact with SOX9 by binding to a conserved GLI-binding element near the SOX9 binding site to repress the expression of *Col10a1* which is a hypertrophic chondrocyte marker [39].

#### Chondrocyte hypertrophy and survival

SOX9 is also required for chondrocyte hypertrophy. Inactivation of *Sox9* in flat chondrocytes near the prehypertrophic chondrocytes results in absence of hypertrophy, enhanced apoptosis and absence of *Col10a1* expression [40]. Additionally, SOX9 prevents premature activation of prehypertrophy and subsequent acquisition of an osteoblastic fate. This is achieved in part through inhibition of *Runx2* expression and β-catenin signaling [30]. SOX9 is also necessary to maintain proper homeostasis of cartilaginous tissues in adult mice [41].

Although *Sox9* RNA is turned off abruptly when chondrocytes undergo hypertrophy, SOX9 protein outlives its mRNA and is necessary for chondrocyte hypertrophy [30, 40]. SOX9 interacts with AP-1 (activator protein 1) family members such as JUN and FOSL2 and they directly co-bind at target motifs to promote transcription of hypertrophic genes in the early hypertrophic chondrocytes [42]. Upon hypertrophy and as SOX9 levels decline, FOXA (forkhead box A) competes off SOX9 to accesses the regulatory elements of target genes such as *Col10a1,* to promote terminal chondrocyte maturation together with RUNX2/3, AP1 and MEF2 (myocyte enhancer factor 2) [43].

#### SOX9 targets classification in chondrocytes

ChIP-Seq analysis shows that SOX9 targets in rat proliferating/early prehypertrophic growth plate chondrocytes comprise all major cartilage ECM genes and many cartilage-specific regulatory genes, including the SOX trio target genes [44]. Two distinct categories of SOX9 targets have been identified in mouse chondrocytes [45]. Class I targets are non-chondrocyte-specific genes such as Kelch-like family member 21 (*Klhl21*), solute carrier family 29 member 1 (*Slc29a1*) and RNA binding motif protein 25 (*Rbm25*), which control basic cellular functions, such as translation, non-coding RNA metabolic process and protein folding. SOX9 associates with basal transcriptional components around the transcriptional start sites (TSS) of these genes. Class II targets are chondrocyte-specific genes in which a SOX9 homodimer directly binds to evolutionarily conserved active enhancers and super-enhancers. Moreover, the abundance of binding sites for AP-1, NFAT (nuclear factor of activated T-cells), FOX, RUNX, and HOX (homeobox) transcription factor families near the SOX trio motifs implies their interactions are required to regulate enhancer activities in chondrocytes [45].

### Testis

#### Testis organogenesis and SOX9 expression

Fetal testicular development in mice is depicted in Fig. S1B. In mice, *Sox9* expression is detected in both XY and XX gonads at very low levels at E10.5. Once the Y-linked sex-determining gene *Sry* is expressed in the developing XY gonad, *Sox9* is strongly up-regulated by E11.5 in the bipotential supporting cells which differentiate into pre-Sertoli cells. In contrast, in the developing XX gonad *Sox9* is down-regulated and the supporting cells differentiate into pre-granulosa cells [46]. *Sox9* then continues to be expressed in Sertoli cells throughout testis development and initiates a cascade of gene interactions and cellular events that drive testis differentiation [47]. Sertoli cells start to proliferate and migrate to surround the germ cells and release signals that are required for the differentiation of fetal Leydig cells (FLCs) and peritubular myoid cells (PMCs) [48]. By E13.5, the male gonad is composed of well-defined testis cords and interstitial space. The testis cords comprise germ cells surrounded by Sertoli cells, PMCs and ECM to provide structural support. The interstitium contains the FLCs which will secret testosterone for virilization. Between E13.5 and E14.5, germ cells in the XY gonad undergo mitotic arrest, whereas germ cells in the XX gonad enter meiosis triggered by retinoid acid [49, 50].

#### Role of SOX9 in fetal testis development

Heterozygous mutations in human *SOX9* lead to Campomelic dysplasia (CMPD), with associated partial or complete XY sex reversal in around 75% of cases [2, 3, 51]. Thus, SOX9 is an important factor during human testis determination. In mice, conditional homozygous inactivation of *Sox9* in the XY gonads results in complete male-to-female sex reversal. *Sox9* knockout males show lack of Sertoli cells and testis cords, and Leydig cells. Also, *Sox9* mutant XY gonads show up-regulation of the ovarian markers *Wnt4* (Wnt family member 4) and *Foxl2* (forkhead box protein L2), and the presence of meiotic germ cells [5, 52]. These data demonstrate that SOX9 is essential for Sertoli cell differentiation and testis determination in mice [5, 52]. SOX9 directly regulates the expression of many target genes in Sertoli cells that have important functions during sex determination and differentiation such as *Ptgds* (prostaglandin D2 synthase), *Fgf9* (fibroblast growth factor 9), *Amh* (anti-Müllerian hormone), *Dhh* (desert hedgehog), *Cyp26b1* (cytochrome P450 family 26 subfamily B member 1), *Sox8*, *Sox9*, and *Foxl2.* These and other genes are discussed below, classified into groups according to their cellular function.

##### Repression of the ovarian pathway

The ectopic expression of the ovarian fate markers *Wnt4* and *Foxl2* [53] in *Sox9* knockout gonads suggests that SOX9 prevents granulosa cell differentiation. Sertoli cell differentiation is promoted by repressing these genes. SOX9 inhibits *Wnt4* via FGF9. In XY gonads, SOX9 upregulates *Fgf9* transcription [54], and it does so directly [55]. In turn, FGF9 represses *Wnt4* transcription, as shown by ectopic addition of FGF9 to XX mouse gonadal cultures [54]. In contrast, ChIP-Seq experiments show that SOX9 binds to the *Foxl2* promoter in the fetal mouse testis, and SOX9 can reduce the basal activity of the *Foxl2* promoter in a mouse Sertoli cell line MSC1 cells [56]. SOX9 also binds to the *FOXL2* locus in the bovine testis, supporting a direct repression of *Foxl2* by SOX9 [55].

##### Activation of the *SoxE* genes *Sox8* and *Sox10*

*Sox8* and *Sox10* are potential target genes of SOX9 as they are down-regulated in *Sox9* knockout mice [52, 55]. *Sox8* and *Sox9* double-mutant mice show more severe phenotypes than *Sox8* or *Sox9* single knockouts during testis differentiation. This implies that they act redundantly to trigger and maintain the expression of downstream genes in the testis [57]. Similarly, SOX10 can activate transcriptional targets of SOX9, and ectopic expression of *Sox10* in the developing XX gonads can cause sex reversal. This explains its ability to direct male development [58]. ChIP-Seq demonstrates that SOX9 binds to the *Sox8* promoter in the fetal mouse testis, and SOX9 can up-regulate endogenous *Sox8* expression and transactivate the *Sox8* promoter in MSC1 cells [56]. SOX9 also binds to the *SOX8* locus in the bovine testis [55], indicating a direct regulation of *Sox8* by SOX9. ChIP-Seq also shows SOX9 binding at the *Sox10/SOX10* loci in the fetal mouse and bovine testis [55, 56].

##### SOX9 expression maintenance

Continuous and sufficient SOX9 expression is required for Sertoli cell differentiation and testis development. There are at least three ways to maintain SOX9 expression in Sertoli cells. The cell-autonomous *Sox9* auto-regulation via SOX9 and steroidogenic factor 1 (SF1), the FGF9/FGFR2-mediated pathway, and the PTGDS/Prostaglandin D2 (PGD2)-mediated pathway. SOX9 maintains its own expression by directly regulating the expression of *Ptgds*, *Sox9* (see “Regulation of the *SOX9*/*Sox9* gene”) and possibly of *Fgf9*. In XY *Sox9* knockout mice, *Ptgds* expression is almost absent [59]. Conversely, XY *Ptgds* knockout gonads show reduced *Sox9* expression levels and a delay in testicular differentiation [59]. In vitro studies show that SOX9 can transactivate the *Ptgds* promoter through binding to a paired SOX-site within the promoter of *Ptgds* as a homodimer [60]. ChIP assays confirm that SOX9 can also bind to the paired SOX binding sites in the *Ptgds* promoter in vivo. These findings imply that *Sox9* and *Ptgds* form a positive feed-forward loop which maintains *Sox9* expression in the XY gonads. SOX9 is essential for *Fgf9* expression. In XY *Sox9*^−/−^ gonads at E11.5, *Fgf9* expression is reduced or absent [54]. In turn, FGF9 is necessary for maintaining *Sox9* expression in Sertoli precursor cells at E12.5. Thus, *Sox9* and *Fgf9* generate a positive feed-forward loop in XY gonads and both genes are required for somatic cell proliferation. Support for *Fgf9* being a direct target of SOX9 comes from the SOX9 ChIP-Seq studies in mouse and bovine testes, which show that SOX9 binds to the *Fgf9/FGFG9* locus in both species [55].

##### Sertoli cell driven germ cell and Leydig cell differentiation

In XY *Sox9* knockout testes, germ cells are undergoing meiosis, and Leydig cells do not form, like in XX gonads [5, 52]. SOX9 might directly control *Cyp26b1* and *Dhh* gene expression in Sertoli cells to regulate the fate of germ cells and Leydig cell differentiation, respectively. CYP26B1 prevents premature entry of XY germ cells into meiosis through degradation of retinoic acid [50]. In *Sox9* or *Sf1* conditional knockout XY gonads, *Cyp26b1* expression is significantly decreased at E13.5, indicating that SOX9 and SF1 are involved in positive regulation of *Cyp26b1* expression [61]. Indeed, SOX9 and SF1 can synergistically up-regulate endogenous *Cyp26b1* expression in the mouse Leydig cell line TM3 in a dose-dependent manner. In addition, SOX9 can potently activate the *Cyp26b1* promoter in MSC1 cells and binds to the *Cyp26b1* promoter in vivo in the fetal mouse testis [56]. SOX9 also binds to the *CYP26B1* locus in the bovine testis. These data support a direct role for SOX9 in the regulation of the *Cyp26b1* gene.

In humans, mutations in *DHH* gene cause 46, XY gonadal dysgenesis [62]. DHH promotes fetal Leydig cell differentiation and regulates peritubular myoid cell differentiation and male fertility in the mouse [63]. SOX9 together with SF1 can strongly activate the *Dhh* promoter in MSC1 cells, and SOX9 binds to the *Dhh* promoter in the fetal mouse testis [56]. ChIP-Seq also shows that SOX9 binds to the *DHH* locus in the bovine testis [55], supporting a direct regulation of *Dhh* by SOX9.

Cerebellin 4 precursor (*Clbn4*) is also likely to be target gene of SOX9 in the fetal testis, but its testicular function is yet to be elucidated. CBLN4 may direct the differentiation of other cell types in the developing testis as a paracrine factor produced by Sertoli cells [64]. *Cbln4* expression is reduced in XY *Sox9*-deficient mice at E13.5, whereas overexpression of *Sox9* in XX mice upregulates *Cbln4* expression. ChIP analysis shows that SRY and SOX9 can bind to the *Cbln4* enhancer fragment both in vitro and in XY gonads at E11.5 in vivo, indicating a joint function for the regulation of *Cbln4* male-specific expression [64].

##### AMH and Müllerian duct regression

The Müllerian ducts develop into the fallopian tubes, the uterus, and part of the vagina in females. AMH is secreted by Sertoli cells in the male gonads, causing the regression of the Müllerian ducts. There is strong evidence that SOX9 directly regulates the *Amh* gene to regress the Müllerian ducts in males. In XY *Sox9* knockout mice, the Müllerian ducts show no signs of degeneration at E15.5, consistent with the absence of AMH protein expression at E13.5 [5]. Arango et al. [65] have shown that when the SOX9-binding site within the mouse *Amh* promoter is homozygously mutated, *Amh* transcription is not initiated, resulting in males with both male and female internal genitalia. In addition, ChIP-Seq demonstrates that SOX9 binds to the *Amh/AMH* locus in mouse and bovine testes, supporting a direct regulation of *Amh* by SOX9 [55].

#### Adult Sertoli cell function and spermatogenesis

To illustrate the role of SOX9 after the sex determination stage, *AMH-Cre;Sox9*^*flox/flox*^ mice were generated with conditional inactivation of *Sox9* in Sertoli cells at E13.5 [57]. *AMH-Cre;Sox9*^*flox/flox*^ show normal fetal testis development but progressive seminiferous tubule and spermatogenesis defects, and infertility at about 5 months. Two likely SOX9 target genes with important roles mainly during spermatogenesis are *ETV5* (Ets variant gene 5) and *GDNF* (glial cell-line derived neurotrophic factor). ETV5 is a transcription factor that plays an important role in maintaining fertility. Mice lacking *Etv5* lose maintenance of spermatogonial stem cell self-renewal, leading to progressive germ cell loss and a Sertoli-cell-only syndrome [66]. In XY *Ck19-Cre;Sox9*^*flox/flox*^ knockout mice, *Etv5* expression is decreased. Furthermore, ChIP assays show that SOX9 binds directly to a conserved SOX-binding site within the regulatory region of *ETV5* [67]. GDNF is a growth factor produced by Sertoli cells which regulates the cell fate decision of spermatogonia and spermatogonial self-renewal [68]. *Gdnf* expression is reduced in XY *Sox9* knockout gonads [57] and ChIP-Seq on fetal mouse testes identified that both SRY and SOX9 bind to the *Gdnf* promoter [55, 56].

### Nervous system

#### CNS development and SOX9 expression

The central nervous system (CNS) develops from a neural plate that folds to form a neural groove and eventually the neural tube (Fig. S1C). Neural stem cells (NSCs) are a subtype of progenitor cells in the nervous system that can differentiate into both neurons and glial cells. Oligodendrocytes and astrocytes are the two main types of glial cells in the CNS. Different types of neurons and glial cells are generated in different domains of the ventricular zone. pMN and p2 domains first generate motoneurons and V2 interneurons, respectively, and later there is a switch to the generation of oligodendrocytes and astrocytes, respectively.

In mice, *Sox9* is weakly expressed in cells of the ventral neural tube at E9.5 [69]. At E10.5, SOX9 expression is detectable in a few SOX2-positive neuroepithelia throughout the ventricular zone and increases in many cells over the next 24 h [69]. Concurrent with this, multipotent NSCs appear in large quantity after E10.5 [6]. At E12.5, SOX9-positive oligodendrocyte progenitors appear at the border of the ventricular zone, indicating a switch from neurogenesis to gliogenesis [69]. The number of SOX9-expressing cells increases in the surrounding parenchyma while decreases in the ventricular zone, accordant with the reduction of the ventricular zone and neuroepithelial cells. *Sox9* expression is also detected in the migrating, actively proliferating oligodendrocyte progenitors, but downregulated in oligodendrocytes at the onset of terminal differentiation [69].

#### Induction and maintenance of neural stem cells

SOX9 is required for induction and maintenance of neural stem cells [6]. Loss of *Sox9* in the CNS from E11.5 can significantly reduce neurosphere formation which is a widely used in vitro culture system to quantitate the frequency of NSCs. In contrast, overexpression of *Sox9* can induce precocious neurosphere formation from embryonic CNS. In addition, the removal of SOX9 leads to NSCs loss of multipotency.

#### Glial specification

SOX9 is required for the switch from neurogenesis to gliogenesis [69]. Specific deletion of *Sox9* from E10.5 in the CNS in *Nestin-Cre;Sox9*^*flox/flox*^ mice causes a reduction in oligodendrocyte precursors at E12.5, whereas the number of motoneurons is increased. In addition, at E14.5 and E16.5, neural tissue in *Sox9* null mice contains virtually no astrocytes, but more V2 interneurons are present compared with controls [6]. These data indicate that the generation of oligodendrocyte precursors from the pMN domain and astrocyte precursors from other domains of the ventricular zone including the p2 domain is disrupted in the absence of *Sox9*, and that stem cells in these domains continue to produce neurons.

#### Astrocyte differentiation

After glial specification, *Sox9* expression is maintained in the astrocyte lineage [69]. SOX9 induces the crucial transcription factor NFIA (nuclear factor-I A) by binding to its enhancer element and then cooperates with this factor to mediate the initiation of gliogenesis [70]. After glial initiation, SOX9 and NFIA form a complex and cooperatively activate a set of astrocytic genes. These include *Apcdd1* (APC down-regulated 1), *Mmd2* (monocyte to macrophage differentiation associated 2) and *Zcchc24* (zinc finger CCHC-type containing 24), by binding to their promoter regions. Additionally, SOX9 positively regulates the expression of *Nfe2l1* (nuclear factor erythroid-2-like 1), a transcription factor gene that promotes glial maturation, by binding to its promoter in vitro [71]. Thus, SOX9 not only plays a role in astrocyte specification, but also in its differentiation and maturation.

#### Oligodendrocyte differentiation

During oligodendro-gliogenesis, SOX9 and NFIA are required for oligodendroglial specification in the pMN domain of the ventricular zone [70]. After specification, *Sox9* is down-regulated in the oligodendrocyte precursors emigrating from the pMN domain. SOX9 is important for oligodendroglial specification and SOX10 is essential for oligodendrocyte terminal differentiation [72]. Intriguingly, there is a time between specification and terminal differentiation when these two SOXE proteins can work together to regulate *Pdgfra* (platelet-derived growth factor receptor A) for cell survival, proliferation and migration [73].

### Retina

#### Retina development and SOX9 expression

Mouse retina development begins at around E9.5 when the early retina is populated by a pool of multipotent retinal progenitor cells (RPCs) (Fig. S1D) [74]. Six types of neurons and one type of glial cell within the retina arise from the RPCs. During retinogenesis, ganglion cells are first to differentiate with Müller glial (MG) cells the last.

*Sox9* is expressed in the proliferating, multipotent progenitors throughout retinogenesis and then exclusively in Müller glial cells and retinal pigment epithelia (RPE) until adulthood [7, 75]. At E11.5, *Sox9* is expressed in the progenitors at a low level in the center of the retina, while highly in the RPE [74, 75]. At E13.5, *Sox9* expression is clearly observed within the neuroblastic layer of RPCs, and gradually increased in the proliferating RPCs until the postnatal day 1 (P1). From P3, some RPCs exit cell cycle and begin to differentiate, and *Sox9* is down-regulated in the differentiating populations but remains in Müller glial cells and RPE [7, 75].

#### SOX9 functions

In mice with retinal-specific inactivation of *Sox9* starting from E10.5 (*Chx10-Cre;Sox9*^*flox/flox*^), MG cells are absent in *Sox9*-deficient retinal areas, as evidenced by loss of MG markers p27^Kip1^ and glutamine synthetase (GS) [7]. In addition, knockdown of *Sox9* in retina organ cultures prepared from E17.5 mouse embryos reduces the number of MG cells and increases the relative proportion of rod photoreceptors, indicating that SOX9 is required for MG cell specification [75].

Considering continuous *Sox9* expression in the RPE, SOX9 is important for RPE differentiation and maturation [76, 77]. RNA-Seq analyses of mouse RPE reveal that genes related to visual perception and transporter activity are up-regulated while genes related to cell proliferation are down-regulated at P5 compared to E15.5 [76]. When *Sox9* is inactivated in the developing RPE in *Dct-Cre;Sox9*^*flox/flox*^ mice from E11.5, RPE maturation related genes are down-regulated, indicating that SOX9 plays a role in regulating the late stages of RPE differentiation [76, 78]. Furthermore, *Sox9* is expressed in the nuclei of mature RPE cells and regulates the visual cycle genes during postnatal stages [77].

Although *Sox9* expression is not detected in choroid, SOX9 is essential for choroid vasculature development via regulating angiogenesis genes such as vascular endothelial growth factor A (*Vegfa*) and angiopoietin-like 4 (*Angptl4*) in RPE [76]. During choroid development, *Vegfa*, *Vegfb* and *Angptl4* expression is progressively increased in the mouse RPE and also in RPE generated from human embryonic stem cells [76]. In contrast, angiogenesis genes are down-regulated in the developing RPE when *Sox9* is conditionally deleted, leading to poor vasculature and little pigmentation [76, 78].

#### SOX9 targets

SOX9 regulates genes of the visual cycle, as specific deletion of *Sox9* in mouse RPE leads to decreased expression of several visual cycle genes, especially *Rpe65* (retinal pigment epithelium 65) and *Rgr* (retinal G protein-coupled receptor) [77]. Promoter-luciferase assays reveal that SOX9 and OTX2 (orthodenticle homeobox 2) synergistically activate the human *RPE65* and *RLBP1* (retinaldehyde binding protein 1) promoters. Moreover, SOX9 and OTX2 bind to the promoter regions of *RPE65*, *RLBP1*, and *RGR* in bovine RPE and human fetal RPE cells. SOX9 ChIP-Seq analysis in differentiated hES-RPE revealed that several SOX9 target genes are involved in the early stages of age-related macular degeneration (AMD) [76]. Seven out of these eight genes were also downregulated in the *Dct-Cre;Sox9*^*flox/flox*^ mouse. ChIP-PCR assays elucidated that SOX9 and PAX6 (paired box protein 6) cooperatively bind to the first exon of *ANGPTL4* in hES-RPE [76].

### Lung

#### Lung development and SOX9 expression

The lungs and trachea arise from the anterior foregut endoderm (Fig. S1E). Morphogenesis of the respiratory system begins around E9.0 when the transcription factor Nkx-2.1 is expressed in a subset of the ventral foregut endoderm cells in mice. By E9.5, the Nkx-2.1-positive epithelial cells evaginate the surrounding mesenchyme to form two primary lung buds and the trachea. Between E9.5 and E12.5, the primary lung buds continue to proliferate and invade the mesenchyme. The trachea gradually separates from the esophagus. During the pseudoglandular stage of fetal lung development (E12.5–E16.5), the primary lung buds undergo branching morphogenesis to generate a tree-like network of airways. Two distinct progenitor cell lineages develop along the proximal–distal axis with SOX2 marking the proximal progenitors, while SOX9 and ID2 (inhibitor of DNA binding 2) marking the distal progenitors. The proximal progenitors give rise to airway neuroendocrine cells, secretory cells, ciliated cells and mucosal cells. The distal progenitors give rise to type 1 and type 2 alveolar epithelial cells (AEC1 and AEC2). During the canalicular (E16.5–E17.5) and saccular (E18.5–P5) stages, branching morphogenesis ceases and the terminal branches expand to form epithelial sacs which later develop into alveoli.

*Sox9* expression can be detected in the distal epithelial progenitors during branching morphogenesis (E11.5–E16.5) in mice. After E16.5, *Sox9* is down-regulated, coincident with the initiation of AEC1 and AEC2 differentiation [8]. *Sox9* is also expressed in a population of mesenchymal cells, which surround the trachea, bronchi, and bronchioles, and that will develop into the future cartilage rings [79].

#### SOX9 functions

SOX9 is essential for branching morphogenesis and alveolar epithelial differentiation [8, 80]. SOX9 functions downstream of FGF10/FGFR2b to promote branching and suppress premature initiation of alveolar differentiation. Specific deletion of *Sox9* using *Shh-Cre* in the lung epithelium before E12 leads to smaller lungs and fewer and dilated airway branches [80]. Conversely, *Sox9* overexpression leads to large and cyst-like structures at the distal epithelial branch tips [8]. Microarray analysis of control and *Sox9* mutant lungs identifies several genes that are down-regulated upon *Sox9* deletion, such as Clusterin (*Clu*) and Melanoma inhibitory activity (*Mia*) which are related to lung development. *Fgf10* expression is up-regulated in the *Sox9*-deficient lung, implying that SOX9 controls epithelial feed-back regulation of *Fgf10* in the mesenchyme. Moreover, SOX9 prevents premature differentiation of the lung epithelium. Inactivation of *Sox9* causes precocious differentiation of AEC2, whereas *Sox9* overexpression inhibits terminal differentiation of distal lung progenitors [8]. Genes expressed by differentiated alveolar cells, such as *Sftpb* (surfactant protein B), *Sftpc* (surfactant protein C), *Lamp3* (lysosomal associated membrane protein 3), *Napsa* (napsin A aspartic peptidase), and *Ctsh* (cathepsin H), are up-regulated in *Sox9*-deficient lungs [80]. These results suggest that SOX9 functions to maintain the undifferentiated status of distal lung progenitors.

SOX9 also plays multiple roles in regulating proliferation, differentiation, ECM organization, cell polarity and migration during lung development. Deletion of *Sox9* using *Shh-Cre* results in cytoskeletal disorganization, ECM defects, and abnormal epithelial movement [8]. Microvilli are common extensions of the apical surface of epithelial cells and are uniform in size and shape. *Sox9*-mutant epithelial cells show reduced or absent microvilli and a rounded apical surface [8]. The control epithelial cells have a flat and uniform basal surface, while the *Sox9*-mutant epithelial cells have a rounded and irregular basal surface. Strikingly, E-cadherin immunofluorescence shows that SOX9 does not affect apical-basal cell polarity [8, 80]. SOX9 also regulates ECM genes in the lung epithelium. Two ECM proteins, COL2A1 and Laminin, are disrupted when *Sox9* expression is altered. SOX9 directly regulates *Col2a1* by binding to its consensus binding site, and loss of *Sox9* leads to reduced expression of *Col2a1/COL2A1* mRNA and protein in lungs [8]. *Sox9* knockout and *SOX9*-overexpressing lungs show mis-localized and reduced Laminin staining at the basement membrane, respectively. However, these defects may be indirect, as no *Laminin* mRNA levels change is found by qRT-PCR. In addition, *Sox9*-deficient epithelial cells show reduced cytoskeleton marker acetylated tubulin along the basal surface. The ECM and microtubule dynamics are strongly associated with cell migration. In vitro assays of E12.5 isolated lung epithelial buds demonstrate that loss of *Sox9* indeed inhibits cell migration [8]. Taken together, SOX9 plays multiple roles in regulating proliferation, differentiation, ECM organization, cell polarity and migration during lung development.

#### SOX9 targets

Based on the SOX9 functions mentioned above, the potential SOX9 targets in the lung could be grouped into branching-related genes and alveolar epithelial differentiation genes [80]. However, these potential SOX9 targets are only inferred from the microarray expression analyses of the control and *Sox9* knockout fetal lungs. Whether SOX9 binds to the regulatory regions of these potential targets has yet to be demonstrated. Another group of SOX9 targets in the lung are ECM genes such as *Col2a1* and *Laminin* [8], which are considered SOX9 targets in various tissues.

### Heart valve

#### Heart valve development and SOX9 expression

Heart valves arise from endocardial cushions in the atrioventricular canal (AVC) and outflow tract (Fig. S1F). Endocardial cushion formation is initiated with endothelial-to-mesenchymal transformation (EMT) of endocardial cells and expansion of the ECM called cardiac jelly. The newly transformed mesenchymal cells continue to proliferate, differentiate and remodel to eventually form the mature heart valve leaflets and supporting chordae tendineae. *Sox9* is directly activated in endocardial cells by Notch signaling which induces EMT at E9.5 in mice during early valve development [80]. By E12.5, SOX9 is highly expressed in the newly transformed mesenchymal cells throughout the developing valves [9].

#### SOX9 functions

SOX9 is essential for endocardial cushion formation and maturation. Mice with germline inactivation of *Sox9* show hypoplastic endocardial cushions whose mesenchymal cells fail to complete EMT, and mice die at E11.5–E12.5. This indicates that SOX9 plays a role in the early stage of endocardial cushion formation [9]. This is further validated in *Tie2-Cre;Sox9*^*flox/flox*^ mice, with conditional inactivation of *Sox9* in endocardial mesenchymal cells. The *Sox9* knockout mice die between E11.5 and E14.5, and manifest with hypoplastic endocardial cushions, reduced proliferation of endocardial mesenchymal cells and altered ECM deposition [81]. In addition, *Col2a1-Cre;Sox9*^*flox/flox*^ mice in which *Sox9* is inactivated in a subpopulation of valve cells during later stages of remodeling die at birth. Mutant mice show thickened valves, reduced expression of cartilage-associated proteins and abnormal ECM patterning [81]. Together, these studies support the idea that SOX9 is required early in valve development for expansion of the heart valve progenitor cell pool after initiation of EMT, and later for normal expression and organization of the ECM.

#### SOX9 targets

SOX9 binds to the promoters or potential regulatory regions of proliferation genes including *Cops5* (COP9 signalosome subunit 5)*, Junb, Fosl1, Fosl2, Fos, Srpk2* (SRSF protein kinase 2)*, Akt2, Eed* (embryonic ectoderm development)*, Hdac1* (histone deacetylase 1), and *Hdac2* in AVC and limb at E12.5, as identified by ChIP-Seq [82]. This illustrates the common function of SOX9 in regulating cell proliferation across different cell types and many developing tissues. In mice, SOX9 is also needed for expression of *ErbB3* (erb-b2 receptor tyrosine kinase 3)*,* as shown by the lack of *ErbB3* expression in *Sox9*-deficient endocardial mesenchymal cells [9]. ERBB3 is a member of the epidermal growth factor receptor tyrosine kinase family and is required for endocardial cushion mesenchymal cell proliferation and myocardium differentiation [83]. Loss of *Sox9* led to altered ECM deposition and organization. SOX9 controls the expression of *Hapln1* (hyaluron and proteoglycan link protein 1)*, Acan, Col2a1, Eln* (elastin)*, Postn* (periostin) and other collagens in the developing valve [82]. ChIP-Seq and RNA-Seq analyses of control and *Tie2-Cre;Sox9*^*flox/flox*^ mice identified several transcription factors as SOX9 targets that participate in heart valve development [82], including *Mecom* (MDS1 and EVI1 complex locus protein) [84]*, Lef1* (lymphoid enhancer binding factor 1) [85]*, Pitx2* (pituitary homeobox 2) [86]*, Hand2* (hand and neural crest derivatives expressed 2) [87]*, Twist1* (twist-related protein 1) [88] and *Sox4* [89]. Among these, SOX9 directly regulates the expression of *Mecom* by binding to its enhancer [82]. The ChIP-Seq study also determined other SOX9 AVC-specific targets, which are categorized into genes involved in DNA binding, cardiac neural crest cell development, and ascending aorta morphogenesis.

SOX9 can also act as a transcriptional repressor during valvulogenesis. Using ChIP-Seq and RNA-Seq, 58 genes were identified as being both bound and repressed by SOX9 in the E12.5 heart valve, including *Junb, Fos, Bhlhe40* (basic helix-loop-helix family member e40) and *Ddit3* (DNA-damage inducible transcript 3). The authors further demonstrated that SOX9 inhibited the activity of the *Junb* promoter in vitro. Another study showed that SOX9 binds and represses transactivation of the osteogenic glycoprotein *Spp1* (secreted phosphoprotein 1) in maturing heart valves and chondrocytes to prevent pathologic matrix mineralization [90].

### Pancreas

#### Pancreas development and SOX9 expression

Mouse pancreas development begins when two dorsal and ventral buds of the foregut endoderm emerge at the boundary between the stomach and duodenum at around E9 (Fig. S1G). During the primary transition (E9.5-E12.5), pancreatic progenitors rapidly proliferate and increase the size of the two buds, where tubulogenesis and patterning occur concomitantly. The two pancreatic buds fuse into a unified organ at the end of the primary transition. The pancreatic epithelium is segregated into two domains. The distal tip domain gives rise to endocrine, ductal and acinar cells while the proximal trunk domain forms ductal and endocrine cells. During the secondary transition (E13.5-birth), the pancreatic epithelium undergoes branching morphogenesis. Simultaneously, the differentiation of endocrine and exocrine cells take place. By the end of the secondary transition, the pancreas has acquired the topological organization of the mature organ while functional maturation continues postnatally.

SOX9 is first detected at E9 and colocalizes with the pancreatic progenitor marker PDX1 (pancreatic and duodenal homeobox 1, also known as MODY4) within the foregut endoderm [10]. From E10.5 to E12.5, *Sox9* remains strongly expressed in all PDX1 + pancreatic epithelial cells, showing that *Sox9* is expressed in pluripotent pancreatic progenitors. At E14, *Sox9* expression continues in a subpopulation of PDX1 + epithelial cells located in the central epithelial cords. At E15.5, *Sox9* expression is restricted to a subset of ductal epithelial cells while absent from committed endocrine progenitors. From E16.5, *Sox9* expression is significantly downregulated and remains exclusive to a small subset of the ductal cells and centroacinar cells from E18.5 to adulthood. SOX9 expression is also detected in human pancreas during embryogenesis [91]. *SOX9* is strongly expressed in the pancreatic primordium at 32 days post-conception (dpc) and remains high at later embryonic stages (52 dpc). Following embryogenesis, SOX9 expression declines in the fetal pancreas and particularly in the islets at 10 weeks gestation. From 14 weeks gestation on, SOX9 is substantially down-regulated and absent from the developing islets. This implies that mouse and human *Sox9/SOX9* show a similar expression pattern during pancreatic endocrine differentiation.

#### SOX9 functions

SOX9 maintains pluripotent pancreatic progenitors by stimulating their proliferation and survival and is also required for the generation of endocrine progenitors [92]. Endocrine and exocrine progenitors are specified within the SOX9-positive epithelial cords. In *Pdx1-Cre;Sox9*^*flox/flox*^ mice with *Sox9* inactivated in pancreatic progenitors, the pancreas displays a reduced PDX1 + progenitor cell pool attributable to reduced cell proliferation and increased apoptosis at E11.5 [10]. In the absence of SOX9, pancreatic cells show reduced *Hes1* (hairy and enhancer of split-1) expression at E11.5, an important marker of multipotent, Notch-responsive and exocrine-restricted progenitors [93]. When *Pdx1-Cre* mediates deletion of a single *Sox9*^*flox*^ allele, mice show about 50% reduction in islet mass, while the islet architecture and the exocrine compartment is normal at E18.5 [92]. This shows that SOX9 is required for the specification of endocrine progenitors, whereas it is dispensable for the generation of exocrine progenitors. Another study shows that *Sox9* haploinsufficiency causes 50% reduced β-cell mass at birth and glucose intolerance during adulthood [94]. This is consistent with a previous study of CMPD patients with heterozygous *SOX9* mutations who show abnormal islet cell morphology and reduced expression of hormone and β cell markers [91]. These findings indicate that SOX9 plays a similar role during pancreas development in humans and mice.

SOX9 is essential for repressing hepatic and intestinal genes in the pancreatic domain. Microarray analysis on E12.5 *Pdx1-Cre;Sox9*^*flox/flox*^ and control pancreata shows up-regulation of α-fetoprotein (*AFP*) and *Albumin*, markers of liver cells, verified by qRT-PCR analysis. This indicates that *Sox9* ablation results in a pancreas-to-liver fate conversion [95]. Conditional deletion of *Sox9* at E11.5 [96] also induced a pancreatic-to-hepatic fate switch, suggesting that the competence window for pancreatic-to-hepatic fate switch in *Sox9*-deleted progenitors closes by E12.5 [95].

Primary cilia are cellular organelles extending from the apical membrane of epithelial cells. In the pancreas, cilia are found in duct and islet cells and are essential for pancreatic organization and function. Loss or disruption of cilia causes ductal abnormalities, polarity defects and dysregulated β-cell function [97]. Pancreatic primary cilia are markedly reduced in *Sox9*-deficient embryos and adults, showing that SOX9 is essential for primary cilia formation in the pancreas [98].

#### SOX9 targets

SOX9 maintains both the pancreatic progenitor identity and expansion through a FGF10/FGFR2b/SOX9 feed-forward loop [95]. Mesenchymal FGF10 binds to FGFR2b of pancreatic progenitors and activates *Sox9* expression, which in turn, activates expression of *Fgfr2b*, thereby maintaining FGF10 receptivity of pancreatic progenitors.

SOX9 activates the pro-endocrine gene *Neurog3* (neurogenin 3) cell-autonomously in bipotent progenitors which can develop either into endocrine cells or ductal cells [98]. NEUROG3 in turn represses *Sox9*, committing the bipotent progenitors to the endocrine program. ChIP assays confirmed that SOX9 binds to the *Neurog3* promoter in both E15.5 mouse pancreata [92] and the mouse pancreatic duct cell line mPAC L20 [99].

SOX9 and PDX1 work together to activate a transcription factor network for pancreatic development and repress an intestinal program by co-occupying the regulatory sequences of particular transcription factor genes [100]. ChIP-Seq analysis of pancreatic progenitors derived from human embryonic stem cells (hESCs) showed that SOX9 and PDX1 are both recruited to regulatory regions of pancreatic transcription factor genes. These include *PTF1A* (pancreas associated transcription factor 1a)*, PAX6*, and *NEUROG3* and several intestinal transcription factor genes, such as *CDX2* (caudal type homeobox 2)*, ONECUT-2* (one cut homeobox 2) and *NKX6-3*. Transcriptional profiling of pancreatic progenitors from compound *Pdx1;Sox9* heterozygous mutant mice shows up-regulation of *Cdx2*, *Onecut-2* and *Nkx6.3*, suggesting direct repression of intestinal cell fate regulators by PDX1 and SOX9 [100].

Following *Sox9* deletion in mouse pancreatic progenitors by E10.5, PDX1 protein expression appears normal at E10.5 [10] but is dramatically down-regulated at E12.5 [94]. This suggests that SOX9 is required for the maintenance of *Pdx1* expression. Conversely, there was no apparent change in SOX9 protein expression in the E10.5 *Pdx1*-deficient pancreas [95]. Therefore, neither PDX1 nor SOX9 is required for the initiation of each other’s expression, but there is evidence that they mutually reinforce expression of each other directly as PDX1 occupies SOX9 regulatory sequences and vice versa [100]. Thus, positive autoregulation of *Pdx1* [101] and *Sox9* [99] and the positive cross-regulatory loop between *Pdx1* and *Sox9* [94, 95] strengthen pancreatic development.

### Bile duct

#### Bile duct development and SOX9 expression

Hepatocytes and biliary epithelial cells (cholangiocytes) arise from the same progenitors, hepatoblasts, during liver development (Fig. S1H). SOX9 expression is first detected in the hepatoblasts near the portal vein mesenchyme at E11.5 in mice [11]. These SOX9-positive hepatoblasts differentiate into cholangiocyte precursors and form the ductal plate which is composed of a single layer of SOX9-expressing cells encompassing the branches of the portal vein. SOX9 expression is then restricted to cholangiocytes. Therefore, SOX9 is the earliest marker of the biliary cell lineage. At E15.5, the ductal plate gradually forms the primitive ductal structure (PDS) which comprises the portal layer of SOX9-positive cholangiocytes and the parenchymal layer of SOX9-negative hepatoblasts [11]. The PDS matures to bile ducts which are surrounded by ECM and mesenchyme, and all cells lining the ducts become typical cholangiocytes.

#### SOX9 functions

SOX9 controls the timing of bile duct morphogenesis during development. Homozygous inactivation of floxed *Sox9* alleles driven by *Alfp1-Cre* (*Alfp1-Cre;Sox9*^*flox/flox*^) in the mouse liver starting at E11.5 leads to delayed maturation of the PDS into bile ducts [11]. The expression of Laminin α5, which is secreted by cholangiocytes and needed for bile duct maturation, is absent in the *Sox9*-deficient liver [102]. Since *Sox9* inactivation only causes mild and transient biliary defects, other members of the SOX family may compensate for the loss of SOX9. Combined ablation of *Sox4* and *Sox9* in the developing mouse liver shows significantly perturbed biliary differentiation both prenatally and postnatally [102]. In contrast, the loss of either *Sox4* or *Sox9* only induced delayed biliary differentiation, and cholangiocyte differentiation returned to normal after birth. Additionally, both *SOX4* and *SOX9* are required for establishing polarity of cholangiocytes. Inactivation of *Sox4* causes loss of apico-basal polarity both at prenatal and postnatal stages, whereas inactivation of *Sox9* results in the absence of polarization only at the fetal stage, and polarization is progressively restored after birth. Notably, *Sox4*;*Sox9* double knockout mice show major polarity defects and irregular cell shape. The apical pole of cholangiocytes has a primary cilium. This functions to detect stimuli from bile and transmit signals into cells regulating several signaling pathways that are required for biliary development such as Notch, TGF-β and Hippo pathways. *Sox4*;*Sox9* double knockouts show perturbed cilia formation leading to a reduced number of cilia after birth. In contrast, deletion of either *Sox4* or *Sox9* display normal cilia formation [102].

SOX4 and SOX9 cooperate to control the signaling pathways TGFβ and NOTCH which are involved in biliary development. Transforming growth factor β receptor II (TβRII) transmits mesenchymal TGF-β signals to hepatoblasts which differentiate into the cholangiocyte lineage, and ΤβRII then becomes repressed in cholangiocytes during duct maturation [11]. At E15.5, TβRII is repressed on the portal side of PDS in wild type but still detectable in all PDS cells in the absence of SOX4 and/or SOX9. At E18.5, TβRII is absent in wild-type cholangiocytes but still detectable in a subset of duct cells in the absence of *Sox4* and in *Sox4;Sox9* double knockout mice [102]. The NOTCH target *Hes1* is not affected at E15.5 but is down-regulated in cholangiocytes at E18.5 in the absence of SOX4 and/or SOX9.

#### SOX9 targets

In *Sox9* conditional knockout mice studies potential SOX9 targets may include genes associated with cholangiocyte differentiation and maturation [102]. RT-qPCR results of the control and *Sox9* knockout liver show that the expression of the apical markers Osteopontin (*Spp1*) and Mucin-1 (*Muc*) is controlled by SOX9 and SOX4, respectively. In addition, SOX9 and SOX4 conjointly affect several signaling pathways controlling biliary development.

### Intestine

#### Intestine development and SOX9 expression

The intestine develops from a flattened, tubular structure, and by E9.5 the gut tube is fully formed and becomes pseudostratified (Fig. S1I). Between E9.5 and E13.5, the intestinal epithelia undergo rapid proliferation, resulting in elongation and increase in circumference and lumen size. From E14.5, the villus morphogenesis begins to form villi and crypt structures. This is followed by epithelial cytodifferentiation into absorptive enterocytes, mucus-producing goblet cells and hormone-producing enteroendocrine cells at around E16.5 [103]. In the small intestine, the antimicrobial agents-secreting Paneth cells settle at the bottom of the crypt as terminally differentiated cells [12]. The mature intestinal epithelium can be divided into a proliferative compartment comprising the crypt of Lieberkühn, and a differentiated compartment comprising the villus in the small intestine and the luminal surface in the colon.

During intestinal development in the mouse, *Sox9* is expressed in all epithelial cells at E13.5 [12]. At E15.5, *Sox9* expression is detected in the rapidly proliferating progenitors, and gradually becomes restricted to intestinal stem cells, transit amplifying progenitor cells, Paneth cells, and enteroendocrine cells by adulthood.

#### SOX9 functions

Differential SOX9 levels mark two distinct populations of *Sox9*^*HI*^ and *Sox9*^*LO*^ cell types in the crypts [104]. *Sox9*^*HI*^ cells represent mature enteroendocrine cells whereas, *Sox9*^*LO*^ cells represent proliferative progenitor cells. Paneth cells also express a high level of SOX9 [105]. Overexpression of *Sox9* in an intestinal progenitor cell line blocks proliferation and induces a transition from an epithelial cell morphology to a neuroendocrine morphology [104]. Moreover, in *Sox9*-deficient mice the proliferative compartment expands to occupy the whole crypt base, leading to crypt hyperplasia throughout the small intestine [106]. These data indicate that a low level of SOX9 promotes progenitor proliferation, whereas a high SOX9 level promotes cell maturation.

SOX9 is essential for the differentiation of Paneth cells and goblet cells [12, 106]. Specific deletion of *Sox9* in mice using *Villin-Cre* (*Vil-Cre;Sox9*^*flox/flox*^) from E10.5 leads to a decreased number of both the Paneth and goblet cells, whereas the enterocyte or enteroendocrine cell populations are unaffected. In addition, overexpression of human *SOX9* upregulates several Paneth cell markers such as *LYZ* (lysozyme), *MM7* and *ANGPT4 *in vitro.

#### SOX9 targets

SOX9 maintains intestinal progenitor cells likely through direct repression of the differentiation genes *CDX2* and *MUC2* [107]. CDX2 is the master regulator of intestine development. ChIP-Seq analysis of hESC-derived pancreatic progenitors showed that SOX9 and PDX1 directly co-bind and repress intestinal cell fate regulators such as *CDX2*, *ONECUT-2* and *NKX6-3* to inhibit intestinal fate in pancreas [100]. Microarray analysis of E12.5 mouse pancreatic epithelia also identified that SOX9 and PDX1 co-regulate *Cdx2*, *Onecut-2* and *Nkx6.3* [100]. MUC2 is the main intestinal mucin covering the intestinal surface and an in vitro assay showed that SOX9 transcriptionally represses the *MUC2* gene via an intermediate repressor protein in human intestinal adeno-carcinoma cells [107].

### Prostate

#### Prostate development and SOX9 expression

The mammalian prostate arises from the urogenital sinus (UGS), which consists of an inner layer of UGS epithelium and an outer layer of UGS mesenchyme (Fig. S1J) [108]. Prostate development relies on androgen, produced by testes from E13-E14 in mice. Prostate morphogenesis starts as the epithelia buds first appear and penetrate the surrounding mesenchyme at E17.5. Then prostate organogenesis continues through birth and prepubertal stages until reaching maturation during puberty. The budding stage is followed by epithelial outgrowth and branching morphogenesis. During the canalization and differentiation stages, the epithelial cords form the ductal lumen and differentiate into luminal secretory cells and basal cells. The surrounding mesenchymal cells differentiate into smooth muscle and fibroblasts. The mature mouse prostate can be divided into four distinct lobes: anterior, dorsal, lateral and ventral. SOX9 is first detected throughout the UGS epithelium and in the mesenchyme near the epithelium at E15 [109]. When the prostatic buds appear at E17.5, *Sox9* is strongly expressed in the prostatic epithelial cells [13]. During bud elongation, SOX9 expression is mainly found in the epithelium with strongest expression at the tips of the growing bud.

#### SOX9 functions

SOX9 plays an important role in the proliferation and branching activity of the prostate. Prostate specific deletion of *Sox9* in *Nkx3.1-Cre;Sox9*^*flox/flox*^ mice leads to a lack of ventral prostate development and abnormal anterior prostate differentiation [13]. Cell proliferation is significantly decreased in the ventral prostate but not in other lobes in the *Sox9* mutant animals compared with controls at E18.5. During later stages of prostate development, SOX9 appears to be less critical. The anterior, dorsal and lateral prostatic lobes show seemingly normal histological structures and cytodifferentiation. The lack of an abnormal phenotype in the dorsal-lateral buds could be due to the inefficient early *Sox9* inactivation in *Nkx3.1-Cre;Sox9*^*flox/flox*^ mice [13].

To delete *Sox9* throughout prostate development, a tamoxifen (TAM)-inducible *ER-Cre;Sox9*^*flox/flox*^ conditional knockout system was utilized [110]. When *Sox9* is ablated at E14.5, the UGS fails to initiate prostate development. When *Sox9* is abrogated at E16.5, only a few prostate buds form. Thus, SOX9 is required for prostate organogenesis before the onset of androgen exposure at E16.5. However, SOX9 is dispensable for adult prostate maintenance.

*Shh-Cre;Sox9*^*flox/flox*^ mice where *Sox9* is specifically deleted from the UGS epithelia by E16.5 immediate prior to bud initiation, shows that SOX9 is important for bud elongation [109]. The number of prostatic buds is not changed while the length of buds is shortened in *Sox9*-deficient mice. Microarray analysis of the control and *Sox9* mutant mice identified that cell migration is most severely impaired in the *Sox9* knockouts. These results suggest that SOX9 is not required for prostatic bud initiation but is essential for bud elongation via promoting cell migration.

#### SOX9 targets

SOX9 regulates cell proliferation via several genes in the ventral prostate lobe. The expression of *Nkx3.1, Shh, Fgfr2*, and *Spry2* (sprouty RTK signaling antagonist 2) genes are severely reduced in the *Sox9* deficient mice at early stages [13]. Microarray analysis from E16.75 control and *Sox9* mutant UGS epithelia identified a subset of SOX9-dependent genes such as *Cdc42* (cell division control protein 42)*, Barx2* (BARX homeobox 2)*, Cxcr4* (C-X-C chemokine receptor type 4) and *Col14a1* that mediate cell migration during prostatic bud elongation [109].

### Hair follicle

#### Hair follicle development and SOX9 expression

In mice, hair follicle morphogenesis begins with hair placode formation at E14 in response to signals from neighboring epidermal cells and underlying mesenchymal cells (Fig. S1K). The epithelial cells proliferate, invaginate downward into the dermis and induce the formation of the dermal papilla (DP). The epithelial cells in close contact with DP constitute highly proliferative matrix cells at the base. Matrix cells continue to proliferate and, while moving upwards, differentiate into cells to form the hair shaft (HS) and the inner root sheath (IRS). The IRS is surrounded by the outer root sheath (ORS), which is contiguous with the epidermis. In the upper ORS and just below the sebaceous gland (SG), there is a region called bulge which contains hair follicle stem cells (HF-SCs) [111].

In adult mice, hair follicles cycle through phases of growth. In anagen, matrix cells undergo proliferation and differentiation leading to downgrowth of the hair follicle into the dermis and upward growth of the hair shaft. During catagen, the lower part of the hair follicle below the bulge undergoes apoptosis. This causes the retraction of the hair follicle and the dermal papilla to rest beneath the bulge. Finally, during telogen, the hair follicle becomes inactive, and the bulge anchors the old hair (club) before starting a new hair cycle. Hair follicle regeneration involves the stimulation of stem cells residing in the bulge [112]. Some HF-SCs exit the bulge and migrate toward the matrix where they become early progenitor cells and subsequently undergo proliferation and differentiation into a new hair follicle [113]. In mice, *Sox9* expression occurs in hair placodes at the time of induction at E14.5 but later it can only be detected in the ORS and the bulge of the hair follicle [14]. In adult mice, *Sox9* is mainly expressed in the quiescent HF-SCs residing in the bulge [20].

#### SOX9 functions

SOX9 is required for matrix cell proliferation and the maintenance of HF-SCs and ORS. Specific ablation of SOX9 in the epithelial layer of the developing mouse skin, starting at E14.5 and complete by P2, led to dramatically impaired proliferative capacity in the matrix cells [14]. Moreover, *Sox9* mutant skin shows loss of the HF-SC marker CD34, loss of external hair, and acquirement of epidermal characteristics in ORS. To demonstrate whether SOX9 is required for hair placode induction, the upper lips of germline-specific E11.5 *Sox9* knockout embryos were trans-planted under the kidney capsules of nude mice. Histological analysis of the transplants after 10 days showed that hair follicles developed, indicating that *Sox9* is dispensable for early hair follicle formation [14].

SOX9 is critical for initial stem cell specification which occurs during the earliest stages of HF morphogenesis [114]. Furthermore, SOX9-expressing stem cells can contribute to all skin epithelial lineages and sebaceous glands. In the absence of SOX9, the early stem cell population never forms, and HF differentiation, sebaceous gland formation and epidermal wound-healing are severely impaired. Thus, SOX9 is required for the maintenance of ORS identity, the formation of the HF-SCs, the proliferation of matrix cells, and epidermal wound-healing, although SOX9 seems to be dispensable for initial hair induction.

When *Sox9* is conditionally ablated in established adult mouse HF-SCs, they lose stemness. This leads to arrested HF downgrowth as hair cycle proceeds. Moreover, the *Sox9*-deficient HF-SCs showed signs of epidermal differentiation. These results suggest that SOX9 is required for maintaining adult HF-SCs identity [20]. Transcriptional profiling of wild-type and *Sox9*-deficient HF-SCs revealed that *Sox9*-deficient bulge cells lose the expression of HF-SC signature genes while they acquire the expression of some interfollicular epidermis signature genes. Together, these findings suggest that SOX9 is essential for adult HF-SC maintenance and suppression of epidermal differentiation in the bulge niche [20].

#### SOX9 targets

SOX9 plays a role in maintaining HF progenitor/stem cells and cell proliferation. However, little is known about SOX9 targets associated with these functions during fetal HF development. In the adult HF-SCs, SOX9 ChIP-Seq together with RNA-Seq data identified a short list of both SOX9-bound and activated genes. These include TF genes such as *Lhx2* (LIM homeobox 2), *Nfatc1* and *Tcf7l2* (transcription factor 7-like 2), ECM genes such as *Timp2* (TIMP metallopeptidase inhibitor 2), *Wfdc3* (WAP four-disulfide core domain 3) and *Col8a2*, and genes encoding TGF-β/Activin signaling factors such as *Sulf2* (sulfatase 2), *Inhbb* (inhibin subunit beta B) and *Wwp2* [20].

## Mechanisms underlying SOX9 common and unique roles

SOX9 acts commonly in roles like cell-fate determination, progenitor maintenance, and cell proliferation, yet also has unique roles within specific tissue. We propose that the mechanisms underlying the SOX9 common functions include control of broadly expressed genes, *Sox9* autoregulation and common signaling pathways like FGF and Notch signaling.

In this section, we focus on the mechanisms by which the unique roles of SOX9 are achieved among different organs. The specificity of SOX9 roles and functions may be explained by the transcriptional regulation of the *SOX9*/*Sox9* genes, the posttranslational modifications of the SOX9 protein, SOX9 binding partners, and enhancers of target genes.

### Regulation of the *SOX9*/*Sox9* gene

#### Enhancers

The large gene desert region upstream and downstream of *SOX9/Sox9* contains regulatory sequences controlling *SOX9/Sox9* tissue-specific expression patterns. Comparison of human and pufferfish genomic sequences highlighted eight highly conserved sequence elements (E1–E8) between 290 kb 5′ and 450 kb 3′ to *Sox9*. These were tested for enhancer activity in transgenic mice using a lacZ reporter gene [115]. The E3 enhancer, located 251 kb upstream of *SOX9*, drove *lacZ* expression specifically within cranial neural crest cells and the inner ear. The E1 enhancer, located 28 kb upstream of *SOX9*, controls *lacZ* expression in the node, notochord, gut, bronchial epithelium and pancreas. The E7 enhancer, located 95 kb downstream of *SOX9*, regulates *lacZ* expression in the telencephalon and midbrain [115].

Yao et al. [116] could show that the E4 sequence element (termed E250), located 250 kb 5′ to mouse *Sox9*, shows enhancer activity, driving *lacZ* reporter gene expression in condensed prechondrocytes but not later in the developing cartilage. They also identified two additional chondrocyte lineage enhancers. E195, located 195 kb 5′ to *Sox9*, drives *lacZ* reporter gene expression in proliferating chondrocytes. E84, located 84 kb 5′ to *Sox9*, shows reporter activity in differentiated chondrocytes but not in the non *Sox9*-expressing hypertrophic chondrocytes. These data suggests that during cartilage development endogenous *Sox9* expression is regulated at least in part by the sequential activities of enhancers E250, E195 and E84. Moreover, a cartilage-specific enhancer (rib cage-specific enhancer, RCSE) was identified 1 Mb upstream of the *Sox9* TSS, and CRISPR/Cas9-mediated deletion of RCSE leads to smaller rib cages in mice [117].

Microdeletions and translocations define a locus around 1.2–1.5 Mb upstream of *SOX9* associated with Pierre-Robin Syndrome (PRS), a rare congenital condition characterized by a hypoplastic mandible. Several enhancers have been identified in this region which drive reporter gene expression in the fetal mouse mandibles, suggesting that *SOX9*-related PRS is caused by the removal of these enhancers [118, 119].

Gonad-specific enhancers of *SOX9/Sox9* were identified by several studies. The 3.2 kb TES enhancer (Testis-specific Enhancer of *Sox9*), located − 13 to − 10 kb upstream of the *Sox9* TSS, can fully recapitulate the gonadal expression pattern of *Sox9* in the mouse [120]. Both SRY and SF1 bind to TES and drive *Sox9* expression in Sertoli cells. Subsequently, SOX9 replaces SRY and interacts with the core element within TES (TESCO), forming a feed-forward regulatory loop to maintain its transcription level. In addition, four novel gonadal enhancers were identified in mouse, with Enh13 and Enh14 showing strong testicular expression [121]. Enh13 is located 565 kb upstream of *Sox9* and, like TESCO, it is bound by SRY and SOX9 in vivo, suggesting that both TESCO and Enh13 are regulated by SRY and SOX9. CRISPR-mediated deletion of either TESCO, Enh13 or Enh14 in mice showed that Enh13 is a critical enhancer for testicular *Sox9* expression and thus testis development. XY gonads lacking Enh13 only expressed 21% of wildtype *Sox9* levels, leading to complete sex reversal. In contrast, XY gonads lacking TESCO or Enh14 still expressed 45% of or normal wildtype *Sox9* levels, respectively, and developed as normal testes. Enh13 might also be important for human testicular *SOX9* expression since it is located within a 32.5 kb locus, whose removal is associated with XY sex reversal [121]. By analyzing the genome data of DSD patients, three testis-specific enhancers eALDI, eSR-A and eSR-B 5′ of *SOX9* were identified in humans which showed synergistic action to drive *SOX9* expression in the testis [122].

While SOX9 autoregulation in the testis is mediated via TESCO and possibly also Enh13, in most other *Sox9*-expressing tissues SOX9 is directly autoregulated via the enhancer SOM, which is located 70 kb 5′ to *Sox9*. CRISPR-mediated deletion of SOM leads to an average reduction of 18–37% in *Sox9* expression levels in the gut, pancreas, liver, lung, kidney and salivary gland [123].

#### Promoters

In addition to enhancers, other regulatory regions may include promoters, silencers, and 3D chromatin organization. Regulatory elements of the *SOX9*/*Sox9* gene have been expertly reviewed elsewhere [124–127]. In brief, in vitro studies of chondrocytic and mesenchymal cell lines show that within the *SOX9*/*Sox9* proximal promoter, functional binding sites occur for transcription factors hypoxia-inducible factor-1α (HIF1α) [128], Notch signaling mediator RBPJ [129], NF-κB subunit RELA [130], CREB and SP1 [131], bone morphogenetic protein 2 (BMP-2) [132], signal transducer and activator of transcription (STAT3) [133], and Yes-associated protein 1 (YAP1)/TEA domain transcription factor (TEAD) [134]. During testicular development, the *Sox9* proximal promoter located − 193 bp to − 73 bp shows higher activity in the mouse testis than in the ovary and liver at E13.5 [135], implying the action of testis-specific factors. However, this *Sox9* proximal promoter region shows only neural tube activity in vivo [115]. This suggests that important tissue-specific factors act on genomic regions outside the promoter, such as SOX9 and SF1 interacting with enhancers TESCO, eALDI, eSR-A and eSR-B [120, 122].

#### Topologically associating domains (TADs)

Topologically associating domains (TADs) are megabase-sized chromatin domains in which regulatory elements such as enhancers and promoters interact with one another more frequently than outside TADs [127]. TADs are separated by boundaries which harbor binding sites for zinc finger transcription factor CTCF and the cohesin protein complexes. The human and mouse *SOX9*/*Sox9* loci contain two major TADs. One harbors the *SOX9*/*Sox9* gene and the large gene desert that contains many enhancers deleted or duplicated in human disease [136]. The other TAD encompasses the genes *KCNJ2*/*Kcnj2* and *KCNJ16*/*Kcnj16* which is separated from the *SOX9*/*Sox9* TAD by a boundary in the gene desert. By modelling Cooks syndrome, a congenital limb malformation, genomic duplications upstream of *SOX9*/*Sox9* creates a new TAD (neo-TAD) containing both the *Kcnj2* gene and the *Sox9* regulatory region leading to limb malformation due to ectopic *Kcnj2* expression [136]. In silico analysis of chromatin conformation of the *SOX9* locus shows that *SOX9* chromatin folding domains associate with real and putative regulatory elements of *SOX9*. Also, there is conservation of chromatin domain boundaries across multiple cell lines [137]. Detailed studies of *SOX9*/*Sox9* chromatin organization await further chromosome conformation capture methods such as capture Hi-C and 4C-seq.

#### Epigenetic regulation of *SOX9*/*Sox9*

Expression of the *SOX9*/*Sox9* gene may be controlled by epigenetic regulatory mechanisms, such as DNA methylation, histone acetylation and methylation. However, most epigenetic studies of *SOX9*/*Sox9* have been in cancer tissues, rather than during organogenesis. For example, in pancreatic cancer, the NF-κB pathway is highly activated. Demethylation of an adjacent CpG island may enhance binding of NF-κB to the *SOX9* promoter thereby increasing SOX9 expression [138]. Conversely, in bladder cancer [139] and melanoma [140], hypermethylation of the *SOX9* promoter leads to SOX9 silencing.

In cartilage, DNA methylation regulates *Sox9* expression [141]. During articular cartilage development, Wnt signals recruit DNA methyl transferase DNMT3A to the *Sox9* promoter, leading to hypermethylation, and thus SOX9 downregulation in the limb bud mesenchymal cells [142]. In contrast, FGF signals block DNMT3A recruitment to the *Sox9* promoter by promoting phosphorylation of DNMT3A. BMP-2 stimulates *Sox9* expression during chondrogenesis in primary mouse embryo fibroblasts [143]. It increases the association of the transcription factor NF-Y with histone acetyltransferase p300, resulting in a complex binding to the *Sox9* promoter. In addition, BMP-2 induces histone hyperacetylation and hypomethylation at the *Sox9* proximal promoter, suggesting that BMP-2 affects *Sox9* gene transcription through histone modification and chromatin remodeling.

### Posttranscriptional regulation of SOX9

#### microRNA

SOX9 regulation by microRNAs, which act as repressors of gene expression, has been widely studied in different types of cancer, but less so during organ development. miR-124-mediated repression of SOX9 protein is important for neuronal differentiation in the adult mouse brain [144]. Moreover, miR-124 can repress both SOX9 translation and transcription in mouse ovarian cells [145]. Over-expression of miR-145 decreases expression of SOX9 protein and miR-145 inhibition significantly elevates SOX9 protein levels [146]. Thus, miR-145 is likely a key negative regulator of SOX9 during early chondrogenic differentiation. IL-1β-induced miR-101 binds to the 3’UTR of *Sox9* in rat chondrocytes, leading to decreased expression of *Sox9* mRNA and SOX9 protein and degeneration of cartilage ECM [147]. miR-1247 targets a highly conserved sequence in the *SOX9* coding region to repress SOX9 protein expression [148]. miR-495 also directly targets the 3’UTR of *SOX9* in human mesenchymal stem cells, leading to reduced SOX9 protein level and inhibition of chondrogenic differentiation [149].

#### Long non-coding RNA

Long non-coding RNAs (lncRNAs) often modulate gene expression in a tissue-specific manner. lncRNAs exceed 200 nucleotides but are not translated. Interactions between lncRNAs and *SOX9* have been explored in the context of cancers [150]. The regulatory role of lncRNAs in organogenesis are less understood. *LOC102723505* (also termed *ROCR*, regulator of chondrogenesis RNA) may act as an upstream regulator of *SOX9* in chondrocytes [151]. *ROCR* is upregulated during chondrogenic differentiation, and upon depletion of *ROCR* via RNAi, *SOX9* mRNA and SOX9 protein levels are significantly reduced, cartilage-specific genes are downregulated, and ECM formation is perturbed. Microarray analysis of mouse neonatal and adult testes shows that over 3,000 lncRNAs are differentially expressed, with many lncRNAs overlapping or adjacent to important transcription factors in testicular development, such as *Sox9*, *Lhx1* and *Wt1* [152].

### Posttranslational modifications of SOX9

Posttranslational modifications (PTMs) affect the stability, intracellular localization, activity, and partner protein interactions of SOX9. PTMs of SOX9 in chondrogenesis is explored in detail by Lefebvre and Dvir-Ginzberg [125], and evolutionary conservation of PTMs by Vining et al. [153].

#### Serine phosphorylation

SOX9 is phosphorylated at three serines (S64, S181 and S211) by cyclic AMP-dependent protein kinase A (PKA) in chondrocytes [154, 155], and also at S181 by cGKII (cGMP-dependent protein kinase type II) [156]. Phosphorylation of SOX9 enhances its DNA-binding affinity and transcriptional activity on a *Col2a1* chondrocyte-specific enhancer. In addition, the parathyroid hormone-related peptide (PTHrP) signal can increase SOX9 phosphorylation and transcriptional activity in the prehypertrophic zone of the growth plate to inhibit chondrocyte maturation. The same phosphorylated sites of SOX9 have been found in Sertoli cells, in which phosphorylation induces SOX9 nuclear translocation and increases its DNA binding affinity [157]. Notably, phosphorylation of SOX9 on S64 and S181 is required for *Sox9*-*Snail2* interaction to trigger neural crest delamination in the developing chick neural tube [158]. Phosphorylation of S64 and S181 also facilitates SUMO-ylation of SOX9 [158] (see below).

#### Arginine methylation

Coactivator associated arginine methyltransferase 1 (CARM1), a member of the arginine-specific protein methyltransferases (PRTMs), methylates SOX9 at multiple arginine residues within the HMG domain in vitro and in vivo [159]. Methylated SOX9 then dissociates from beta-catenin which increases *Cyclin D1* mRNA expression and thus chondrocyte proliferation. CARM1^−/−^ mouse embryos die immediately after birth, have significantly delayed endochondral bone formation, and inhibited chondrocyte proliferation. These data indicate that SOX9 methylation plays an important role in chondrocyte proliferation.

#### Lysine acetylation and deacetylation

Acetylation and deacetylation of SOX9 affect cartilage-specific gene expression. TIP60 acetylates SOX9 at lysine 61, 253 and 398 residues. This leads to more diffuse nuclear localization of both proteins and subnuclear colocalization [23]. The transcriptional activity of SOX9 on *Col2a1* is enhanced by TIP60. However, this effect is independent of enhanced acetylation of SOX9 by TIP60 as the SOX9 mutants K61/253/398A show enhanced transcriptional activity by TIP60 as well. On the contrary, sirtuin 1 (SIRT1) deacetylates SOX9 and increases its transcriptional activity on *COL2A1* in human articular chondrocytes [160]. A further study reveals that deacetylation of SOX9 by SIRT1 promotes SOX9 nuclear translocation and enhances its binding to a -10 kb *ACAN* enhancer in adult human chondrocytes [161].

#### Lysine SUMO-ylation

Small ubiquitin-like modifier (SUMO)-ylation affects SOX9 transcriptional activity in a context-dependent manner. SUMO-ylation of both SF1 and SOX9 represses their synergistic transcription of the mouse *Amh* reporter gene in vitro but does not affect their DNA binding activity or interaction with each other [162]. The acetylated lysine residues of SOX9 (K61, K253 and K398) are also SUMO-ylation sites [23, 163]. Protein inhibitor of activated STAT (PIAS) proteins, which act as SUMO ligases, increase cellular SOX9 protein levels and SOX9-dependent transcription of a *Col2a1* promoter-enhancer reporter [163]. Conversely, PIAS1 enhances the sumoylation of the mouse SOX9 protein but represses SOX9-dependent transcription of the *Col11a2* enhancer reporter gene [164]. Intriguingly, in *Xenopus*, SUMO-ylation of SOX9 and SOX10 directs inner ear development, whereas non-SUMO-ylation promotes neural crest development [165].

#### Ubiquitination and UFMylation

SOX9 is also subject to ubiquitination, which results in degradation of SOX9 via the ubiquitin-proteosome pathway. E6-AP ubiquitinates SOX9 in vitro and in vivo, and SOX9 may be ubiquitinated by E6-AP in hypertrophic chondrocytes given the opposing distribution of SOX9 and E6-AP: with E6-AP high in hypertrophic chondrocytes, but low in prehypertrophic chondrocytes, and the converse for SOX9 levels [166]. Further, E6-AP-deficient mice have an accumulation of SOX9 in chondrocytes and the brain [166].

On the contrary, UFMylation, a posttranslational modification where a ubiquitin-like fold modifier 1 (UFM1) covalently binds to its targets, prevents SOX9 proteasomal degradation [167]. SOX9 UFMylation is likely regulated by the DDRGK domain-containing protein 1 (DDRGK1). In vitro assay shows that DDRGK1 directly binds to SOX9 and inhibits its ubiquitination and proteasomal degradation. In addition, *Ddrgk1* deficient mice show decreased SOX9 protein levels and chondrogenic mesenchymal condensation. Furthermore, a loss-of-function mutation in the *DDRGK1* gene has been identified in Shohat-type spondyloepimetaphyseal dysplasia (SEMD), a chondrodysplasia characterized by vertebral, epiphyseal, and metaphyseal abnormalities.

### SOX9 and its transcriptional partners

**Fig. 2:**
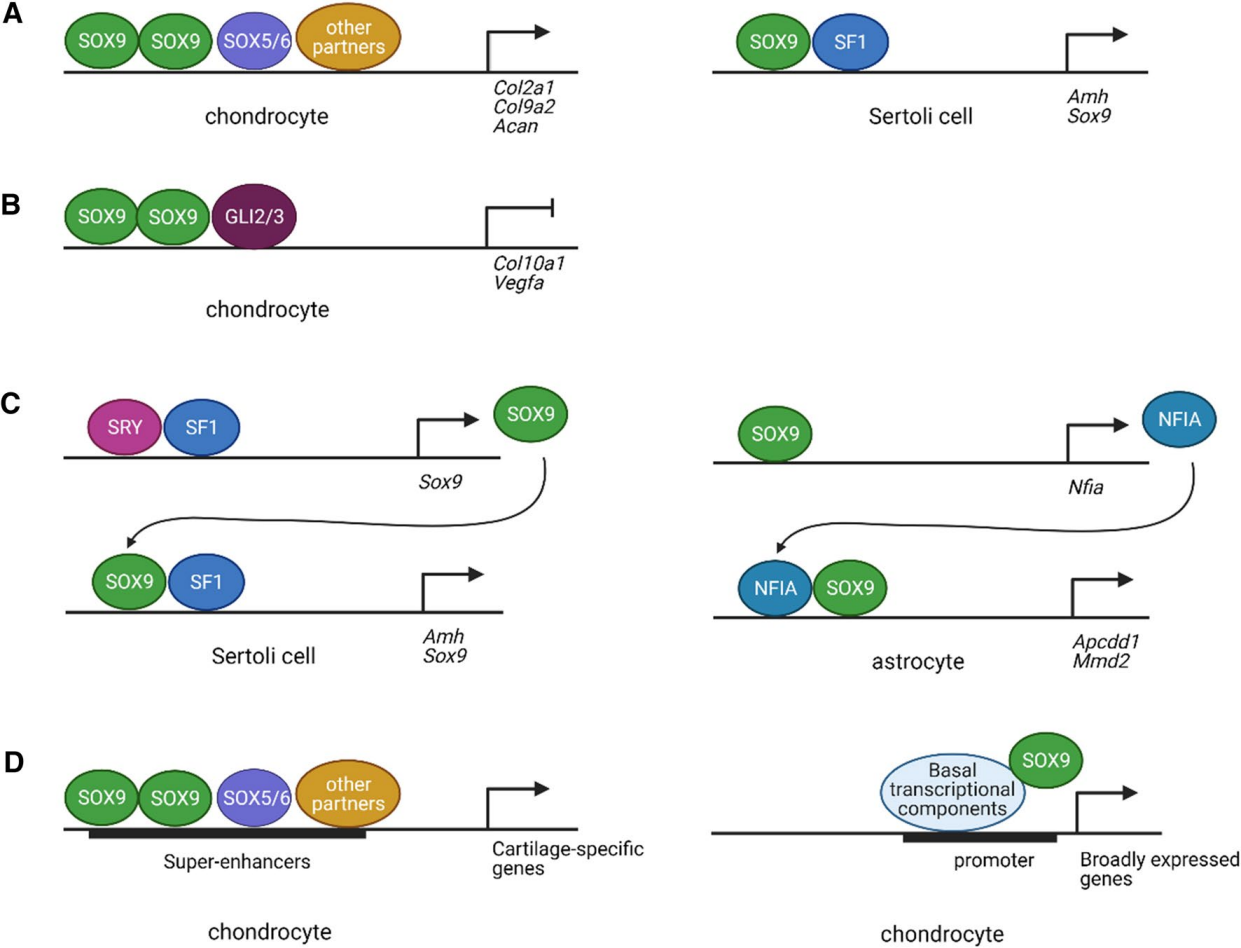
Regulation of target genes by SOX9 and partners. **A** SOX9 homodimer binds to inverted SOX binding sites in multiple enhancers of chondrocyte-specific genes. It physically interacts with SOX5/SOX6 and other partners which bind to DNA sequences close to SOX9 binding sites. SRY and SF1 function together to regulate *Amh* and *Sox9* itself in Sertoli cells. **B** Apart from transactivating targets, SOX9 forms complex with GLI factors to repress genes such as *Col10a1* and *Vegfa*. **C** SOX9 and partner proteins regulate stepwise progression of tissue development. In Sertoli cells, SRY and SF1 induce *Sox9* expression and then SOX9 forms complex with SF1 to promote subsequent gene expression. During astrocyte differentiation, SOX9 induces *Nfia* expression and then interacts with NFIA protein to promote astrocytic gene expression. **D** Multiple TFs bind to super-enhancers to regulate cartilage-specific gene expression in chondrocytes, while SOX9 indirectly engages the genome via interacting with basal transcriptional components to regulate widely expressed gene expression in chondrocytes

SOX9 exhibits diverse functions in a vast number of tissues in part through forming complexes with functional partners to cooperatively activate and/or repress target genes (Fig. 2A). Target genes of SOX9 often harbor binding motifs for SOX9 partner proteins adjacent to the SOX binding sites to facilitate cooperative transcriptional activity. For example, in cartilage, SOX9 homodimer binds to the homodimer-binding sequences on the enhancer regions of chondrogenic genes such as *Col2a1* and *Col11a2*, while SOX5 and SOX6 bind to pairs of SOX motifs close to *SOX9* and potentiate SOX9 transcriptional activity [16, 168]. ChIP-Seq analysis has identified abundant binding sites for AP-1, NFAT, RUNX, HOX and FOX transcription factor families near the SOX trio motifs, indicating multiple transcription factors cooperate to regulate cartilage-specific genes [45]. In the testis, SOX9 and SF1 bind to their respective binding elements and synergistically activate *AMH/Amh* expression [65, 169]. Based on ChIP-Seq and RNA-Seq analyses of murine and bovine fetal testes, it was found that SOX9-bound genomic regions are enriched in binding motifs for three Sertoli cell reprogramming factors SOX9, DMRT1 (doublesex and mab-3 related transcription factor 1) and GATA4 (GATA binding protein 4) [55]. This SOX9, DMRT1 and GATA4 DNA-binding motif enrichment is specific to fetal Sertoli cells and conserved among mammals and thus called Sertoli Cell Signature (SCS). In the pancreas, SOX9 works with PDX1 to activate pancreatic genes by co-binding the regulatory sequences with other pancreatic transcription factors [100].

SOX9 predominantly acts as a transcriptional activator but can also function as a repressor. The transcriptional activation or repression effects appear to be in part modulated by protein partners and co-activators or co-repressors. For instance, SOX9 and SOX5/6 function together to activate ECM genes in early stage and proliferating chondrocytes, whereas SOX9 interacts with GLI factors to repress the transcription of *Col10a1*, a marker for chondrocyte maturation in hypertrophic chondrocytes (Fig. 2B) [39]. Furthermore, SOX9 induces expression of Notch coactivator Mastermind-like transcriptional activator 2 (*MAML2*), which, independently of Notch signaling, can promote beta-catenin turnover [170]—indicating that SOX9 can indirectly work as a repressor via other factors. SOX9 can also bind directly to β-catenin, preventing TCF/LEF-mediated signaling and promoting β-catenin degradation [25].

SOX9 interacts with AP-1 family members such as JUN and FOSL2, and they directly co-bind at target motifs to promote hypertrophic gene expression [42]. In addition, peroxisome proliferator-activated receptor γ co-activator 1α (PGC-1α) directly interacts with SOX9 as a coactivator to directly regulate *Col2a1* transcription by binding to its enhancer during chondrogenesis [171]. SOX9 can regulate expression of *Runx2* via several mechanisms: indirectly via activation of the transcriptional repressor vertebrate homologue of *Drosophila* bagpipe (Bapx1), which negatively regulates expression of *Runx2* [172]; and also directly, via modulating the ubiquitination status of RUNX2, decreasing ubiquitination and redirecting it toward lysosomal degradation [173].

Furthermore, SOX9 enables a stepwise progression of development by forming complexes with different partners or co-factors (Fig. 2C). In the male gonad, SRY and SF1 directly bind to TESCO to induce *Sox9* expression. SOX9 then substitutes SRY, and physically interacts with SF1 to maintain its own expression and promote subsequent developmental processes [120]. SOX9 is required for glial specification and subsequent astrocyte differentiation in the CNS [69]. SOX9 activates the transcription of *Nfia* by binding to its enhancer element and then cooperates with NFIA to promote gliogenesis by inducing a set of astrocytic genes [70].

These data thus highlight the diversity and complexity of the roles of SOX9, directly and indirectly activating or repressing the expression of many target genes to modulate tissue-specific expression profiles.

### Cell-specific enhancers and super-enhancers of SOX9 target genes

Master transcription factors regulate cell identity genes by co-occupying clusters of enhancers, i.e., super-enhancers, which are different from typical enhancers in size, transcription factor binding motif density and content, transcriptional activity, and sensitivity to perturbation [174]. In contrast, transcription factors interact with basal transcriptional components to indirectly bind to promoters of broadly expressed genes. In non-hypertrophic chondrocytes, ChIP-Seq analysis shows that SOX9 and SOX5/6 bind to enhancers and super-enhancers of cartilage-specific genes (Fig. 2D) [44, 45]. These super-enhancers also feature binding sites for multiple transcription factor families close to the SOX trio binding sequences, supporting the notion that cell-specific genes are controlled by several key transcription factors. In mouse HF-SCs, SOX9 was identified as the pioneer factor that promotes and maintains cell fate [175]. Also, SOX9 binds to super-enhancers with multiple transcription factors to regulate cell-specific genes. SOX9 is sensitive to local microenvironment changes and responds to changes by remodeling super-enhancers.

## Conclusions and future directions

The transcription factor SOX9 is critical for mammalian embryonic development. *Sox9* ablation affects the development of multiple organs, such as bone, testis, heart, retina, pancreas, and lung. Investigations into the regulation of the *SOX9/Sox9* gene, posttranslational modifications and partner factors have helped to understand the mechanisms underlying the diverse SOX9 functions in different tissues and at different stages in mammals. In view of the complexity and size of the regulatory regions of *SOX9/Sox9* and numerous target genes, further studies are needed to identify novel regulatory sequences such as enhancers, transcriptional partners and the mechanisms by which SOX9 regulates target genes across different tissues.

## Supplementary Information

Below is the link to the electronic supplementary material.

Supplementary file1 (PPTX 2337 KB)

## Data Availability

Not applicable.
