# Supplementary material for: SOX9 in organogenesis: shared and unique transcriptional functions
Source: Cell Mol Life Sci. 2022 Sep 17;79(10):522. doi: 10.1007/s00018-022-04543-4 (PMC9482574; doi:10.1007/s00018-022-04543-4)
Supplement: Supplementary file 1 — Supplementary file1 (PPTX 2337 KB) [file 18_2022_4543_MOESM1_ESM.pptx]

## Slide 1
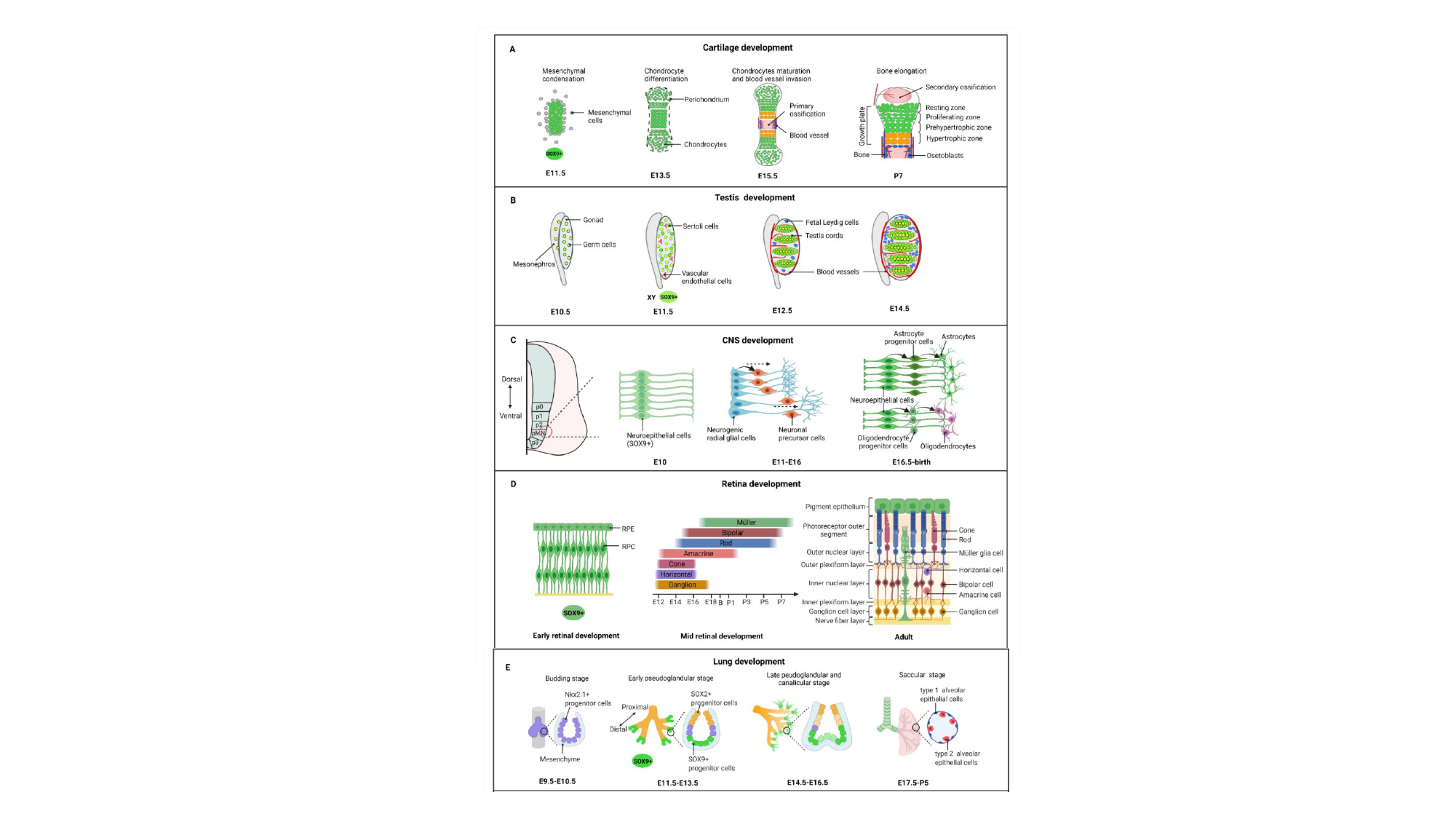

## Slide 2
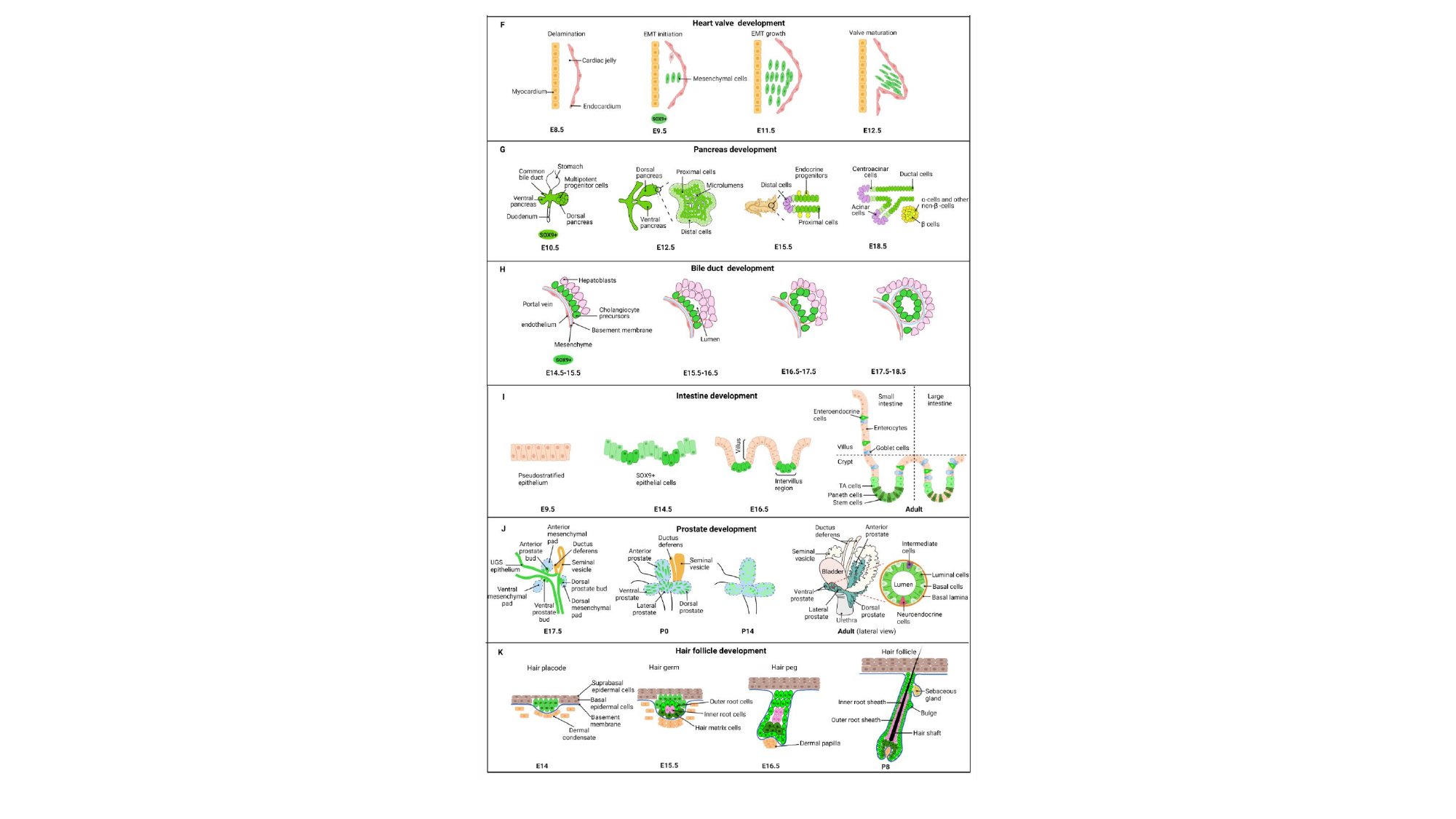

## Slide 3
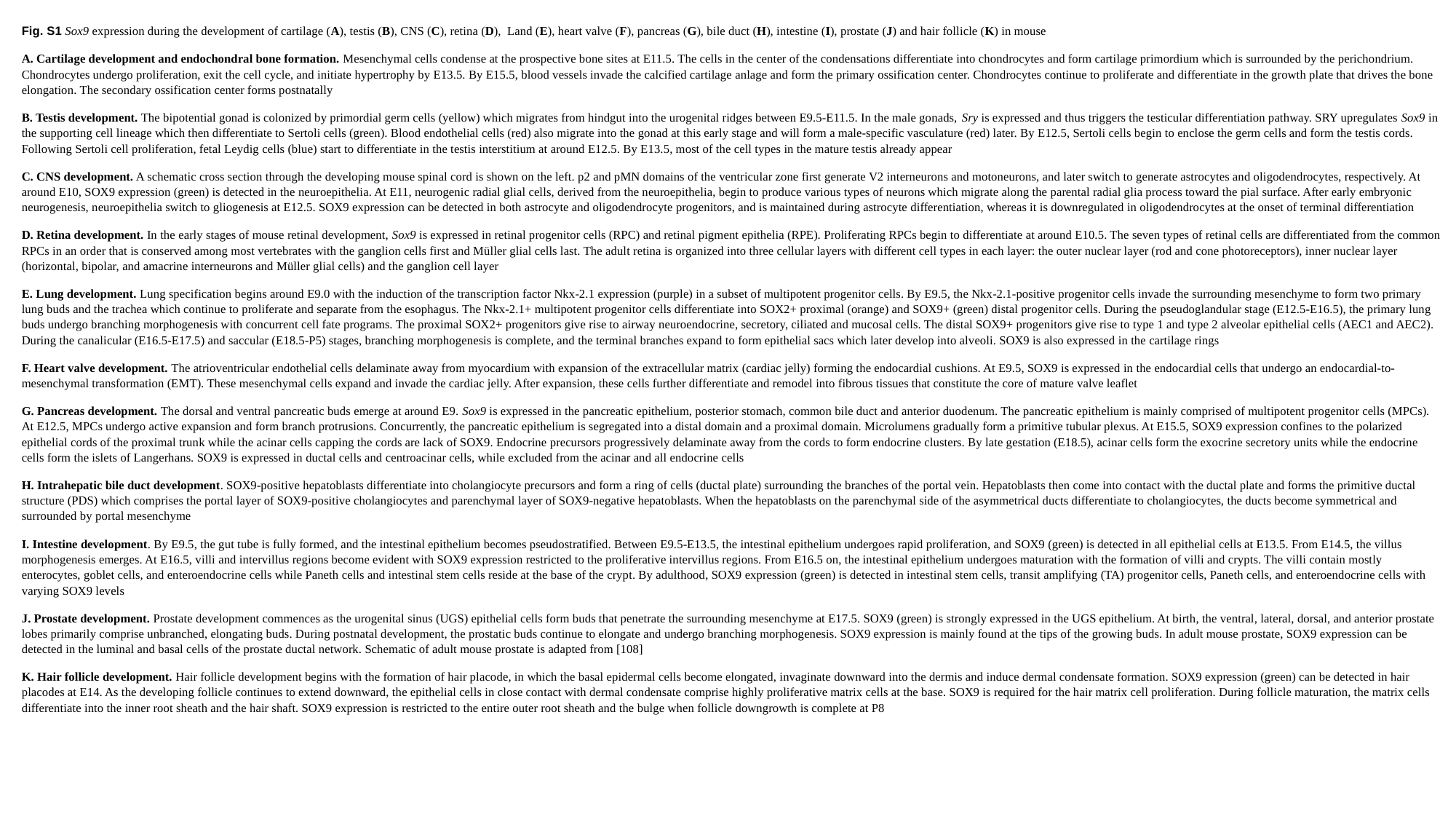

Fig. S1 Sox9 expression during the development of cartilage (A), testis (B), CNS (C), retina (D), Land (E), heart valve (F), pancreas (G), bile duct (H), intestine (I), prostate (J) and hair follicle (K) in mouse
A. Cartilage development and endochondral bone formation. Mesenchymal cells condense at the prospective bone sites at E11.5. The cells in the center of the condensations differentiate into chondrocytes and form cartilage primordium which is surrounded by the perichondrium. Chondrocytes undergo proliferation, exit the cell cycle, and initiate hypertrophy by E13.5. By E15.5, blood vessels invade the calcified cartilage anlage and form the primary ossification center. Chondrocytes continue to proliferate and differentiate in the growth plate that drives the bone elongation. The secondary ossification center forms postnatally
B. Testis development. The bipotential gonad is colonized by primordial germ cells (yellow) which migrates from hindgut into the urogenital ridges between E9.5-E11.5. In the male gonads, Sry is expressed and thus triggers the testicular differentiation pathway. SRY upregulates Sox9 in the supporting cell lineage which then differentiate to Sertoli cells (green). Blood endothelial cells (red) also migrate into the gonad at this early stage and will form a male-specific vasculature (red) later. By E12.5, Sertoli cells begin to enclose the germ cells and form the testis cords. Following Sertoli cell proliferation, fetal Leydig cells (blue) start to differentiate in the testis interstitium at around E12.5. By E13.5, most of the cell types in the mature testis already appear
C. CNS development. A schematic cross section through the developing mouse spinal cord is shown on the left. p2 and pMN domains of the ventricular zone first generate V2 interneurons and motoneurons, and later switch to generate astrocytes and oligodendrocytes, respectively. At around E10, SOX9 expression (green) is detected in the neuroepithelia. At E11, neurogenic radial glial cells, derived from the neuroepithelia, begin to produce various types of neurons which migrate along the parental radial glia process toward the pial surface. After early embryonic neurogenesis, neuroepithelia switch to gliogenesis at E12.5. SOX9 expression can be detected in both astrocyte and oligodendrocyte progenitors, and is maintained during astrocyte differentiation, whereas it is downregulated in oligodendrocytes at the onset of terminal differentiation
D. Retina development. In the early stages of mouse retinal development, Sox9 is expressed in retinal progenitor cells (RPC) and retinal pigment epithelia (RPE). Proliferating RPCs begin to differentiate at around E10.5. The seven types of retinal cells are differentiated from the common RPCs in an order that is conserved among most vertebrates with the ganglion cells first and Müller glial cells last. The adult retina is organized into three cellular layers with different cell types in each layer: the outer nuclear layer (rod and cone photoreceptors), inner nuclear layer (horizontal, bipolar, and amacrine interneurons and Müller glial cells) and the ganglion cell layer
E. Lung development. Lung specification begins around E9.0 with the induction of the transcription factor Nkx-2.1 expression (purple) in a subset of multipotent progenitor cells. By E9.5, the Nkx-2.1-positive progenitor cells invade the surrounding mesenchyme to form two primary lung buds and the trachea which continue to proliferate and separate from the esophagus. The Nkx-2.1+ multipotent progenitor cells differentiate into SOX2+ proximal (orange) and SOX9+ (green) distal progenitor cells. During the pseudoglandular stage (E12.5-E16.5), the primary lung buds undergo branching morphogenesis with concurrent cell fate programs. The proximal SOX2+ progenitors give rise to airway neuroendocrine, secretory, ciliated and mucosal cells. The distal SOX9+ progenitors give rise to type 1 and type 2 alveolar epithelial cells (AEC1 and AEC2). During the canalicular (E16.5-E17.5) and saccular (E18.5-P5) stages, branching morphogenesis is complete, and the terminal branches expand to form epithelial sacs which later develop into alveoli. SOX9 is also expressed in the cartilage rings
F. Heart valve development. The atrioventricular endothelial cells delaminate away from myocardium with expansion of the extracellular matrix (cardiac jelly) forming the endocardial cushions. At E9.5, SOX9 is expressed in the endocardial cells that undergo an endocardial-to-mesenchymal transformation (EMT). These mesenchymal cells expand and invade the cardiac jelly. After expansion, these cells further differentiate and remodel into fibrous tissues that constitute the core of mature valve leaflet
G. Pancreas development. The dorsal and ventral pancreatic buds emerge at around E9. Sox9 is expressed in the pancreatic epithelium, posterior stomach, common bile duct and anterior duodenum. The pancreatic epithelium is mainly comprised of multipotent progenitor cells (MPCs). At E12.5, MPCs undergo active expansion and form branch protrusions. Concurrently, the pancreatic epithelium is segregated into a distal domain and a proximal domain. Microlumens gradually form a primitive tubular plexus. At E15.5, SOX9 expression confines to the polarized epithelial cords of the proximal trunk while the acinar cells capping the cords are lack of SOX9. Endocrine precursors progressively delaminate away from the cords to form endocrine clusters. By late gestation (E18.5), acinar cells form the exocrine secretory units while the endocrine cells form the islets of Langerhans. SOX9 is expressed in ductal cells and centroacinar cells, while excluded from the acinar and all endocrine cells
H. Intrahepatic bile duct development. SOX9-positive hepatoblasts differentiate into cholangiocyte precursors and form a ring of cells (ductal plate) surrounding the branches of the portal vein. Hepatoblasts then come into contact with the ductal plate and forms the primitive ductal structure (PDS) which comprises the portal layer of SOX9-positive cholangiocytes and parenchymal layer of SOX9-negative hepatoblasts. When the hepatoblasts on the parenchymal side of the asymmetrical ducts differentiate to cholangiocytes, the ducts become symmetrical and surrounded by portal mesenchyme
I. Intestine development. By E9.5, the gut tube is fully formed, and the intestinal epithelium becomes pseudostratified. Between E9.5-E13.5, the intestinal epithelium undergoes rapid proliferation, and SOX9 (green) is detected in all epithelial cells at E13.5. From E14.5, the villus morphogenesis emerges. At E16.5, villi and intervillus regions become evident with SOX9 expression restricted to the proliferative intervillus regions. From E16.5 on, the intestinal epithelium undergoes maturation with the formation of villi and crypts. The villi contain mostly enterocytes, goblet cells, and enteroendocrine cells while Paneth cells and intestinal stem cells reside at the base of the crypt. By adulthood, SOX9 expression (green) is detected in intestinal stem cells, transit amplifying (TA) progenitor cells, Paneth cells, and enteroendocrine cells with varying SOX9 levels
J. Prostate development. Prostate development commences as the urogenital sinus (UGS) epithelial cells form buds that penetrate the surrounding mesenchyme at E17.5. SOX9 (green) is strongly expressed in the UGS epithelium. At birth, the ventral, lateral, dorsal, and anterior prostate lobes primarily comprise unbranched, elongating buds. During postnatal development, the prostatic buds continue to elongate and undergo branching morphogenesis. SOX9 expression is mainly found at the tips of the growing buds. In adult mouse prostate, SOX9 expression can be detected in the luminal and basal cells of the prostate ductal network. Schematic of adult mouse prostate is adapted from [108]
K. Hair follicle development. Hair follicle development begins with the formation of hair placode, in which the basal epidermal cells become elongated, invaginate downward into the dermis and induce dermal condensate formation. SOX9 expression (green) can be detected in hair placodes at E14. As the developing follicle continues to extend downward, the epithelial cells in close contact with dermal condensate comprise highly proliferative matrix cells at the base. SOX9 is required for the hair matrix cell proliferation. During follicle maturation, the matrix cells differentiate into the inner root sheath and the hair shaft. SOX9 expression is restricted to the entire outer root sheath and the bulge when follicle downgrowth is complete at P8
